# SEMPAI: a Self‐Enhancing Multi‐Photon Artificial Intelligence for Prior‐Informed Assessment of Muscle Function and Pathology

**DOI:** 10.1002/advs.202206319

**Published:** 2023-08-15

**Authors:** Alexander Mühlberg, Paul Ritter, Simon Langer, Chloë Goossens, Stefanie Nübler, Dominik Schneidereit, Oliver Taubmann, Felix Denzinger, Dominik Nörenberg, Michael Haug, Sebastian Schürmann, Roarke Horstmeyer, Andreas K. Maier, Wolfgang H. Goldmann, Oliver Friedrich, Lucas Kreiss

**Affiliations:** ^1^ Institute of Medical Biotechnology Department of Chemical and Biological Engineering Friedrich‐Alexander University Erlangen‐Nuremberg Paul‐Gordan‐Str. 3 91052 Erlangen Germany; ^2^ Erlangen Graduate School in Advanced Optical Technologies Paul‐Gordan‐Str. 6 91052 Erlangen Germany; ^3^ Pattern Recognition Lab Department of Computer Science Friedrich‐Alexander University Erlangen‐Nuremberg Martensstr. 3 91058 Erlangen Germany; ^4^ Clinical Division and Laboratory of Intensive Care Medicine KU Leuven UZ Herestraat 49 – P.O. box 7003 Leuven 3000 Belgium; ^5^ Department of Radiology and Nuclear Medicine University Medical Center Mannheim Medical Faculty Mannheim Theodor‐Kutzer‐Ufer 1–3 68167 Mannheim Germany; ^6^ Computational Optics Lab Department of Biomedical Engineering Duke University 101 Science Dr Durham NC 27708 USA; ^7^ Biophysics Group Department of Physics Friedrich‐Alexander University Erlangen‐Nuremberg Henkestr. 91 91052 Erlangen Germany

**Keywords:** deep learning, explainable artificial intelligence, meta‐learning, multiphoton microscopy, muscle research, prior information integration, scientific machine learning

## Abstract

Deep learning (DL) shows notable success in biomedical studies. However, most DL algorithms work as black boxes, exclude biomedical experts, and need extensive data. This is especially problematic for fundamental research in the laboratory, where often only small and sparse data are available and the objective is knowledge discovery rather than automation. Furthermore, basic research is usually hypothesis‐driven and extensive prior knowledge (priors) exists. To address this, the Self‐Enhancing Multi‐Photon Artificial Intelligence (SEMPAI) that is designed for multiphoton microscopy (MPM)‐based laboratory research is presented. It utilizes meta‐learning to optimize prior (and hypothesis) integration, data representation, and neural network architecture simultaneously. By this, the method allows hypothesis testing with DL and provides interpretable feedback about the origin of biological information in 3D images. SEMPAI performs multi‐task learning of several related tasks to enable prediction for small datasets. SEMPAI is applied on an extensive MPM database of single muscle fibers from a decade of experiments, resulting in the largest joint analysis of pathologies and function for single muscle fibers to date. It outperforms state‐of‐the‐art biomarkers in six of seven prediction tasks, including those with scarce data. SEMPAI's DL models with integrated priors are superior to those without priors and to prior‐only approaches.

## Graphical abstract

The Self‐Enhancing Multi‐Photon AI (SEMPAI) that is designed specifically for basic laboratory research with microscopy is presented. It allows to integrate hypotheses and uses meta‐learning in a biologically interpretable configuration space for knowledge discovery. SEMPAI is applied to a large database of multi‐photon microscopy images of single muscle fibers to gain a deeper understanding of structure–function relationships and pathologies.

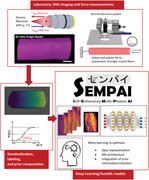

## 1 Introduction

Artificial intelligence (AI) and especially deep learning (DL) is experiencing great success in the classification of digital image data. These algorithms are nowadays regularly used for research in the medical field for automated diagnostics in macroscopic imaging, such as computed tomography (CT) or magnetic resonance imaging (MRI), for example to estimate the effectiveness of radiation therapy,^[^ ^1^ ^]^ to automatically phenotype COPD,^[^ ^2^ ^]^ or to segment organs.^[^ ^3^ ^]^

For fundamental research in the laboratory (lab) and in animal models, however, the automation aspect is much less relevant. Instead, lab research is more concerned with basic discoveries that can lead to a better understanding of pathology or function. Specifically for animal models and fundamental research with microscopy, the mere automation of disease detection does not add much value, since trained DL algorithms require translation to humans. In addition, lab experiments are often of small sample size and labels are sparse, thus rather data‐hungry DL should not be employed to mitigate overfitting, and the lab researcher therefore has to rely on hypothesis‐based research, often in combination with statistics. However, it would be helpful to use hypotheses, while having a system that can recognize patterns independently, e.g., via convolutional neural networks (CNN) that identify relevant features automatically. The field of optical microscopy, in particular, has already benefited from a broad variety of AI applications,^[^ ^4^ ^]^ such as automation,^[^ ^5^, ^6^ ^]^ segmentation,^[^ ^7^ ^]^ and image quality (IQ) enhancement including optimal illumination,^[^ ^8^ ^]^ emitter localization in super‐resolution microscopy,^[^ ^9^ ^]^ or image restoration.^[^ ^10^ ^]^ However, current research mostly covers AI optimization of the microscope settings and is less focused on the hypothesis‐based approach of small‐scale experiments for biological knowledge discovery. A scientist conducting basic research in the lab is also often unfamiliar with the selection of an appropriate DL architecture and associated hyperparameter tuning, which can also be a limiting factor for the use of AI in the lab.

All of these points justify the need for an AI that is designed specifically to meet the needs of a biomedical lab researcher. To understand how this can be achieved, we briefly introduce two cutting‐edge areas of research: meta‐learning and the integration of prior knowledge.

Meta‐learning, or “learning to learn”, analyzes which conditions must be given to be able to effectively learn a specific task. This includes the relatively new field of neural architecture search (NAS),^[^ ^11^ ^]^ with the goal to automatically identify a suitable NN architecture for a given problem. Meta‐learning might replace the time‐consuming trial‐and‐error process of manual architecture search and may not only provide competitive performance, but also solutions with particularly desirable properties, such as curiosity.^[^ ^12^ ^]^ On the downside, NAS, and more generally meta‐learning, are computationally expensive approaches, although a variety of techniques are developed to decrease time and associated costs.^[^ ^13^ ^]^ Recently, a novel meta‐learning approach for segmentation problems in biomedical imaging gained a lot of attention: nnU‐Net^3^. nnU‐Net optimizes NN architecture and hyperparameters together with rule‐based image processing operations (normalization, resampling etc.), with the eponymous U‐Net serving as the base NN architecture. This approach outperformed most prevailing methods for many automated segmentation problems in biology and medicine.^[^ ^3^ ^]^

And although DL has shown its strengths for big data, e.g., for automated classification of images in the world wide web, for fundamental medical research with limited data sets, methods based on prior knowledge can show competitive performance for describing or predicting a pathology.^[^ ^14^ ^]^ Providing prior biological knowledge, or in brief “priors”, to the learning algorithm as a baseline instead of starting from scratch, therefore, seems plausible. Another common drawback of many DL systems is the lack of explainability. A large number of methods, such as DeepSHAP,^[^ ^15^ ^]^ are developed to highlight the image information relevant for the decision‐making process. However, a fundamental question posed by Rudin^[^ ^16^ ^]^ was why the current research focuses on post‐hoc explanations of complicated models rather than creating more interpretable models from the beginning. Explainability can be increased by using priors, such as established measurements or known biomarkers, in the learning process of a NN. Additionally, the integration of prior knowledge in the form of known operators as NN layers was already shown to stabilize the learning process by reducing the maximum error bounds.^[^ ^17^ ^]^ Lastly, by integration of biological priors, human understanding and intuition about a problem can be employed within AI research. Modern AI approaches for microscopy also already started to integrate prior knowledge of imaging physics. For instance, the integration of physics knowledge into the learning process of an AI helped with the technological optimization of microscope‐ and software‐components^[^ ^18^, ^19^ ^]^ for enhanced IQ, and with digital staining of virtual fluorescence in label‐free phase microscopy,^[^ ^20^ ^]^ or Fourier ptychography microscopy.^[^ ^21^ ^]^

Based on the considerations regarding an AI for the lab, and the cutting‐edge areas of meta‐learning and prior‐integration discussed above, we present the Self‐Enhancing Multi‐Photon Artificial Intelligence (SEMPAI) that is specifically designed to integrate hypothesis‐driven priors in a meta‐learning approach for fundamental research. SEMPAI as a general tool simultaneously identifies optimal data representation, degree of prior integration, and NN architecture for a given biomedical problem. In contrast to the technologically‐inspired optimization of microscope parameters for enhanced IQ, it performs biologically‐inspired meta‐learning, i.e., the optimization in a biologically interpretable configuration space, on already existing databases for knowledge discovery. Additionally, SEMPAI utilizes multi‐task learning over different tasks to leverage common patterns shared over all prediction tasks to also enable the prediction for small and sparse data sets. Lastly, SEMPAI's models that are trained on a large joint database with different pathologies in animal models could then be used as foundation models,^[^ ^22^, ^23^ ^]^ and be fine‐tuned for, e.g., translation from ex vivo to in vivo experiments or from animal models to humans. Summarizing, SEMPAI aims to integrate the hypotheses of researchers and identify biologically relevant information in experiments of low sample size, simultaneously serving as a generator of foundation models based on a large database.

To demonstrate the value of this approach, we apply SEMPAI to an extensive and unique multi‐study data collection of 1,298 3D second‐harmonic generation (SHG) images from isolated muscle fibers of different morphologic, genetic, pathologic, or functional conditions. Images of the database were acquired with label‐free multiphoton microscopy (MPM), and functional parameters with highly automated robotized biomechatronics systems.

## 2 Results

### 2.1 SEMPAI Method Overview

In the context of this publication, priors are handcrafted features, i.e., either already known imaging biomarkers or novel features that were developed based on the researcher's hypotheses. Labels, as usual, define the values to be learned and predicted.

**Figure 1:**
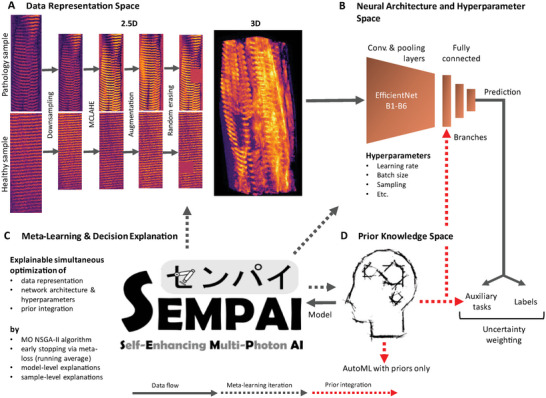
SEMPAI method overview. For each iteration, termed trial, of the self‐enhancement process, a data representation (DaRe) is selected that represents the images either in 2.5D, i.e., by three regularly spaced slices, or in 3D. Then, decisions are made regarding the modification of the DaRe such as downsampling or contrast enhancement. The selected DaRes are fed to a NN, and the NN architecture and its hyper‐parameters are selected. The level of prior integration is then chosen. SEMPAI decides, whether priors i) are not employed, ii) are used as auxiliary tasks for the NN training, iii) are fed directly to the fully connected layer of the NN as branches, or iv) are used in both integration methods, i.e., a combination of (ii) and (iii). In option v), the priors are the only input to an AutoML approach for handcrafted features. The resulting model of the trial is used to predict the labels on the dev set. The performance of the model yields the meta‐losses that guide SEMPAI's configuration selection for the next trial. This process results in a simultaneous self‐enhancement of DaRe, NN architecture & hyperparameters and prior integration with increasing number of trials. Table 1 shows the configuration space. Scale bar: 25 µm.

SEMPAI simultaneously optimizes configurations of its three main components: the prior integration, the data representation (DaRe), and NN architecture and hyperparameters (NN settings). This self‐enhancement in a biologically interpretable configuration space is logged, and its evaluation enables knowledge discovery. The method is shown in **Figure** **1**, the configuration space in **Table** **1**, an extended rationale and explanations for the configuration space, as well as details about the implementation, in Methods.

**Table 1:** Configuration space. Decisions made by SEMPAI during self‐enhancement process

**Data Representation Variants**					
**Contrast Enhancement**	*Yes*: the MCLAHE algorithm is applied on the images	*No*: No further enhancement after registration and resampling			
**Down‐sampling**	*Yes*: Images are downsampled to 0.75µm voxel size isotropically	*No*: No resampling, 0.5 µm isotropically			
**Augmentation**	*Yes*: Application of 3D augmentation such as Gaussian noise, rotation, flipping, affine transformation	*No*: Original standardized images are used			
**Random Erasing**	*Yes*: Random regions of the image are erased	*No*: Original standardized images are used			
**Volume/slice selection**	*3D*: The whole 3D array of each sample is used	*2.5D_1*	*2.5D_5*	*2.5D_10*	*2.5D_20*
		Center slice and 2 slices with 1, 5, 10 or 20 µm distance to the center slice are selected			

**Prior Integration Variants**					
**Method**	*NoPriors*	*AuxLosses*	*Branches*	*AuxLosses*&*Branches*	*PriorsOnly*

**NN Settings Variants**		
**Capacity**	2D/3D EfficientNet *B1‐B6*	
**Learning rate**	Cyclic (*Yes/No*) and in range [0.0001, 0.2]	
**Optimizer**	Adam or SGD with Nesterov momentum	
**Momentum**	Momentum in range [0.9, 0.99]	
**Gradient Clipping**	*Yes*: gradients are clipped to the norm 1.0	*No*: NN gradients evolve freely
**Batch Size**	2.5D/3D: *small* (32/4); *medium* (64/8), *large* (128/16), *XL* [256/32], *XXL* (512/64)	
**Imbalance Sampling**	*Yes*: class distributions are re‐balanced based on strata information of the initial train‐dev‐test split. Sampling weights are estimated automatically	*No*: The original data distribution is fed in the NN

SEMPAI can choose from five different levels to integrate priors (or hypotheses). It can learn without priors (*NoPriors*), use them as auxiliary tasks (*AuxLosses*), which results in a soft constraint to the learning problem^1^, integrate them as an additional branch into the fully connected layer of the NN (*Branches*), or a combination of both (*AuxLosses@Branches*). In the fifth configuration (*PriorsOnly*), only priors are used in an integrated AutoML method^[^ ^24^ ^]^ for handcrafted features, i.e., without using the SHG images and DL. To the best of our knowledge, the integration within SEMPAI is the first attempt to combine current priors (biomarkers) known in single fiber muscle research with ML. Further extended explanations are provided in Methods.

The decisions by SEMPAI regarding DaRe indicate “how and where” biological information can optimally be learned. For example, SEMPAI analyzes whether *3D* images are needed or whether three regularly spaced representative slices (*2.5D*) are sufficient and how large this spacing should be. Analogously, SEMPAI provides information on the importance of downsampling, which can help to estimate the required image resolution for a learning task. As a side effect, this feedback may also have an impact on future studies. For instance, if SEMPAI finds that larger pixel sizes are sufficient for a given task, future data could be acquired in shorter scan times, increasing experimental throughput.

For NN training, in addition to hyperparameter optimization, SEMPAI selects one architecture variant from the base architecture EfficientNet^[^ ^25^ ^]^ that offers scaled variants with different capacity (*B1* to *B6)* and for 2D or 3D, and has been shown to yield competitively predictive performance with less DOF than alternative architectures.^[^ ^25^ ^]^ Accordingly, this architecture allows relatively fast training, making it advantageous for utilization with time‐consuming meta‐learning. SEMPAI learns all tasks jointly in a multi‐task setup. Our hypothesis is that this enforces a semantic regularization of the learning process, since systematic differences unrelated to the biological origin, e.g., in IQ, are less likely to be used for prediction. Instead, the use of related muscle‐specific patterns across different learning tasks is enforced. Multi‐task learning further has the advantage that tasks with small data can still be learned, as DOF are determined by information from similar tasks with more data.^[^ ^26^ ^]^ Recent research shows that this joint learning is preferable to the similar concept of transfer learning.^[^ ^27^ ^]^ In case of missing labels for either primary or auxiliary losses of a sample, no backpropagation occurs during training for the corresponding model outputs, i.e., these outputs are “masked” for that sample. This results in a sort of interleaved learning, in which different tasks are learned in different batches. It also enables joint training without the need for data imputation, thereby enabling effective analysis of sparse and heterogeneous laboratory data. During NN training, all losses are weighed against each other by uncertainty weighting.^[^ ^28^ ^]^

Data to be analyzed by SEMPAI are split into training (train), development (dev) and test set (more details in 2.3). The train set is used to train the NN, while the dev set is used to optimize SEMPAI's decisions in the configuration space. The test set remains unseen. The resulting model of each trial created on the train set is applied for prediction of the labels on the dev set. The predictive performance of the chosen model for each task is assessed for the dev set. Those performances are used as meta‐losses to select the configurations for the next trial. SEMPAI uses NSGA‐II^[^ ^29^ ^]^ multi‐objective optimization for this selection, i.e., there is not only one loss to be minimized, but the losses of all labels are minimized independently.

For tasks with small data, i.e., pCa50 and passive force (**Table** **2** in the next Section 2.2), ML and especially DL are severely limited due to overfitting. Based on the identified associations of the same priors with the investigated labels in previous studies,^[^ ^30^, ^31^, ^32^, ^33^, ^34^ ^]^ we hypothesize that muscle‐specific learning tasks are related and the mean predictive performance over all tasks may assist SEMPAI to select an even more regularized model. Therefore, a *total meta‐loss* is introduced, which is a weighted sum of all meta‐losses for each task and provides an estimate of the model performance over all tasks. This loss is not used for optimization, but for selection of models for small data tasks (N<100).

**Table 2:** Used multi‐study data after standardization and exclusion of data with inappropriate IQ. Please note that the total number of unique images is not a sum of all above, since most images had information for multiple labels, e.g., an image from *mdx*, where active force was available. Example images for each of the included studies are shown in Supporting Information 1. WT: wild type, C: classification, R: regression

Label / Task	Data set with reference	Total number of curated images
Inflammatory phenotype: Sepsis/WT, C	A^[^ ^30^ ^]^	731
Dystrophic phenotype: *mdx*/WT, C	B1^[^ ^31^ ^]^, B2^[^ ^31^ ^]^, C^[^ ^31^ ^]^, D^[^ ^32^ ^]^	567
Muscle type: Diaphragm/EDL, C	D^[^ ^32^ ^]^	179
Active Force, R	B1^[^ ^31^ ^]^	232
Active Force/pCa, R	B1^[^ ^31^ ^]^	152
Passive (Restoration) Force, R	C^[^ ^31^ ^]^	39
pCa50, R	B2^[^ ^31^ ^]^	39
**Total number of unique images**		**1,298**

SEMPAI explains itself regarding i) decision‐making during the self‐enhancement process (SEMPAI model‐level explanations) as well as regarding ii) the decision‐relevant image pixels/voxels and priors of each sample (SEMPAI sample‐level explanations): i) For each task, based on the performed experiments and their results, SEMPAI retrospectively fits a random forest model to estimate its predictive performance from a given configuration. Subsequently, the fitted model is fed in the SHAP Tree Explainer^[^ ^35^ ^]^ to estimate the impact of DaRe, NN settings, and prior integration and identify configurations that yield models with good predictive performance. ii) For the sample‐level explanation of important image regions, SEMPAI utilizes Deep SHAP.^[^ ^15^ ^]^ In the case of prior integration method *Branches* or combined *AuxLosses@Branches*, the method was extended to provide attribution of priors together with, and orthogonally to, the attribution map of the image.

### 2.2 Database, Priors, and Labels

To apply SEMPAI, a database for an organ has to be fed in. Without compromising the general approach of the method, in this section we present and describe the specific database analyzed by SEMPAI. The standardization of this database is explained in the next Section 2.3.

We retrospectively screened in‐house experimental MPM data of single muscle fibers acquired over more than a decade to obtain a large database that includes a variety of biological properties with respect to muscle pathology and function. For these data, we utilize current MPM imaging biomarkers as priors while pathological and functional parameters serve as labels, which we describe below.

A variety of muscle pathologies affect a structured muscle morphology, leading to reduced function of the entire system. For instance, Duchenne muscular dystrophy (DMD) results in an overall loss of structural integrity in individual fibers, eventually leading to failure of respiratory and heart muscle that can be life‐limiting.^[^ ^36^ ^]^ Besides chronic degenerative diseases like DMD, also acute myopathies can result in disruptions of the myofibrillar structural alignment, as it has been shown in ongoing sepsis.^[^ ^30^ ^]^

Function of muscle tissue is based on its passive mechanical and its active force generation properties. Passive force parameters are related to the visco‐elastic behavior of the muscle. In contrast, active force parameters describe its intrinsic ability to generate force, e.g., represented by the physiological sensitivity to calcium ions. Automated integrated biomechatronics systems, such as the *MyoRobot* ^[^ ^37^, ^38^ ^]^ or the *MechaMorph* system,^[^ ^31^ ^]^ can measure these active and passive parameters simultaneously to imaging of the fiber. Both aforementioned systems consist of force transducers (FT) to measure force and voice coil actuators (VCA) to perform axial movement with higher precision as compared to stepper motors.^[^ ^39^ ^]^ A combination of high‐resolution label‐free SHG microscopy with biomechanical measurements of active and passive force was recently demonstrated.^[^ ^31^ ^]^ Through this, correlations between morphological features derived from SHG and functional properties acquired with FTs and VCAs were experimentally shown for individual muscle fibers from *mdx* and wild type (WT) mice.

Table 2 shows the investigated learning tasks, i.e., the labels, the corresponding original studies, and the number of samples used. The extended variants, i.e., larger sample size, of the following studies are included in our database: A) For investigating muscle atrophy during sepsis, samples from the *extensor digitorum longus* (EDL) of septic and WT mice were imaged and complemented by active force recordings in EDL single fibers of the same animal.^[^ ^30^ ^]^ Sepsis is used here as a surrogate for the inflammatory phenotype. B1) Active force measurement and subsequent SHG imaging at each force recording were carried out in EDL single fibers from WT and *mdx* mice.^[^ ^31^ ^]^ B2) Force recordings from a different image data set of EDL fibers to deduce the Ca^2+^ sensitivity of the contractile apparatus, pCa50, as a measure for the troponin‐C Ca^2+^ sensor characteristics.^[^ ^38^, ^40^ ^]^ C) The same setting was used to access passive force parameters on a different set of animals.^[^ ^31^ ^]^ D) Fixated single fibers and fiber bundles from EDL and diaphragm in *mdx* and WT animals were imaged to investigate structural differences between *mdx* and WT as well as between the muscle types of EDL and diaphragm.^[^ ^32^ ^]^ Here, the *mdx* mice serve as a surrogate for the dystrophic phenotype.

The 3D images presented in this data set were generated by label‐free SHG microscopy. Compared to other, macroscopic label‐free imaging modalities, such as MRI,^[^ ^41^ ^]^ CT,^[^ ^42^ ^]^ or ultra‐sound,^[^ ^43^ ^]^ SHG imaging provides sub‐µm resolution to resolve sarcomeres (≈2 µm in size), which is relevant to establish a deeper understanding of the structure‐function relationship and the impact of pathologies on single muscle fibers.^[^ ^44^, ^45^ ^]^ From these images, morphological image features were computed with previously reported software.^[^ ^46^ ^]^ In brief, these features include the *cosine angle sum* (CAS) taken from selected 2D planes (2D‐CAS) and in 3D (3D‐CAS), the *vernier density* (VD), the 3D sarcomere length (3D‐SL), and the cross‐sectional area (CSA) of single fibers. Since these features have already been shown to be descriptive for a variety of rather specific remodeling patterns in muscle research, related to aging, chronic degenerative or inflammatory myopathies,^[^ ^30^, ^31^, ^33^, ^34^ ^]^ we use them as priors.

A more elaborate explanation of the image acquisition, the robotized biomechatronics system, and the extraction of priors is provided in Methods.

### 2.3 Cross‐Study Data Standardization, Data Split, and Performance Metrics

**Figure 2:**
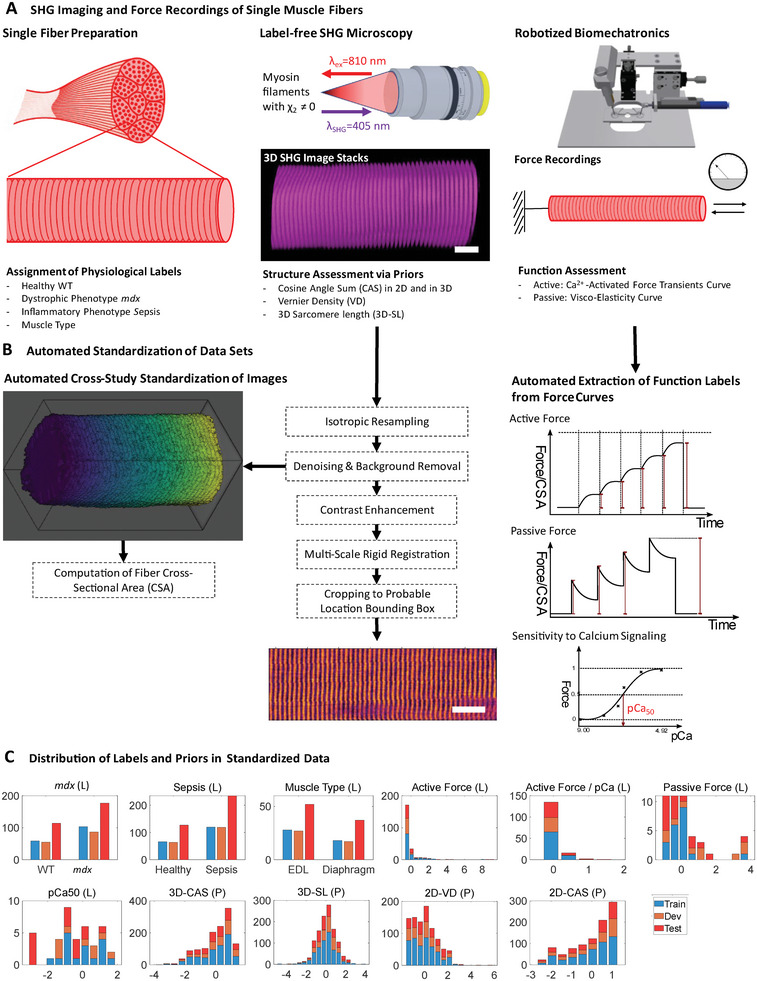
Data acquisition, cross‐study standardization, and value distribution of labels and priors in train, dev, and test set. A) Single muscle fibers were dissected from murine muscle tissue. The data were annotated regarding pathologies and muscle type. In each case, 3D label‐free second harmonic generation (SHG) microscopy was performed, and morphological features, termed priors, were calculated. Muscle tissue was assessed for its function by robot‐assisted biomechanical force measurements. B) The SHG images are standardized with a dedicated image processing pipeline consisting of resampling, denoising, registration, contrast enhancement and cropping of the images to a probable location bounding box. Within this standardization process, the cross‐sectional area (CSA) of the fibers is calculated. Function labels like active force or pCa50 are automatically computed from the raw curves coming from the biomechatronics system. C) Distribution of priors (P) and labels (L) in train, development (dev), and test data after stratified grouped data split. The distributions are normalized to standard score. Scale bar: 25 µm.

The workflow for data acquisition, standardization, and the resulting data distribution after splitting in train, dev, and test set is shown in **Figure** **2**.

The original experiments were conducted by different experimenters, with different imaging systems and parameters, resulting in a high degree of data heterogeneity. Thus, standardization is required to compare images from different studies acquired under varying experimental conditions and during different time periods. By standardization, the technical variance can be minimized. In brief, the images are resampled to an isotropic voxel size of 0.5 µm, slightly denoised via a median filter, and the background, which is defined by Otsu's thresholding of the image, is set to zero. Then, the Multidimensional Contrast Limited Adaptive Histogram Equalization (MCLAHE) algorithm^[^ ^47^ ^]^ is applied for contrast enhancement of each muscle fiber. This contrast‐enhanced image is registered to a pre‐selected fiber with canonical orientation and fiber pattern by a rigid multi‐scale registration, and the resulting transformation is applied to the non‐enhanced version. A mean image of the registered fibers is created after setting all foreground voxels to one, which provides the probability of presence for muscle fibers in each voxel. The bounding box of voxels with probability >0.85 is generated (extent: 180 × 80 × 57 µm^3^) and applied to the images. This cropping of images to relevant regions enables the use of DL models with reduced degrees of freedom (DOF), which is advantageous for our data regime. To allow a uniform comparison of the CSA between each of the individual experiments, we developed a method that combines three different variants of CSA estimation to detect outliers and to be more robust. Further fine‐grained details about the implementation of the standardization and the CSA estimation are provided in Methods.

For our meta‐learning, the standardized data are stratified and grouped into train, dev, and test set (2/4, 1/4, 1/4). The grouping prevents different images of single fibers extracted from the same muscle bundle from being distributed over different sets, which would result in information leakage. The stratification ensures that the distribution in the respective sets is similar, thus, label instances with rare occurrence are present in the train, dev, and test sets. The label and prior data are normalized to a standard score based on mean and standard deviation of the train set. The predictive performance is given for the unseen test set (holdout) as area under the curve (AUC) of the receiver operating characteristic for classification tasks and as R^2^ for regression tasks.

### 2.4 SEMPAI as a Tool for Fundamental Knowledge Discovery

**Figure 3:**
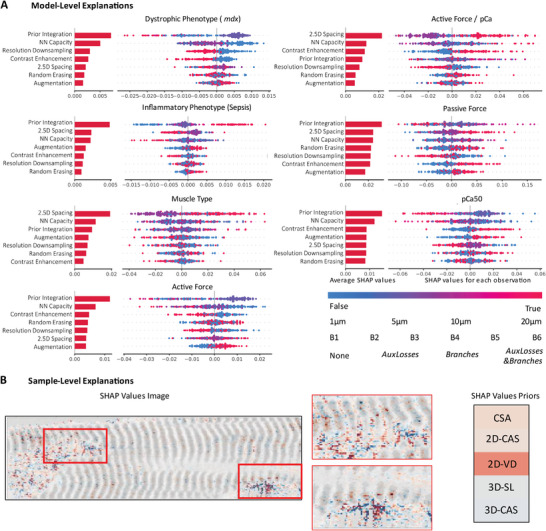
Decision explanation regarding the self‐enhancement process, i.e., model‐level explanations (A), and regarding decision‐relevant image voxels/pixels and priors, i.e., sample level‐explanations (B). A) A random forest model learns the predictive performance of SEMPAI for a specific label as a function of the configuration space. The resulting model is then analyzed by SHAP Tree Explainer that allows to estimate the individual contribution of each configuration for each sample in units of the performance metrics (AUC/R^2^). Decisions are sorted top‐to‐bottom based on their mean absolute SHAP values as a surrogate for the importance of the decision. Configurations are color‐coded from weak to strong expression of a configuration (legend in lower right). B) Attribution map of image (left) and priors (right) for one *mdx* sample. Colored voxels and priors are used by SEMPAI for this sample to correctly predict *mdx*. The attribution of priors is computed simultaneously and shown with the same color code and scale.

As described above, to explain its decision‐making on the model‐level, SEMPAI computed the respective SHAP values of the samples and the mean absolute SHAP values over all samples to quantify the association of the configuration space with the predictive performance. In addition, the stability of the analysis was tested (see Methods). The results are shown in **Figure** **3**A.

In five of seven investigated tasks, the level of prior integration was the most important decision. For the classification tasks *mdx*, sepsis, and muscle type, the integration of prior knowledge (or hypotheses) was especially important according to the mean absolute SHAP values. While *mdx* and muscle type preferred the soft constraint of priors as *AuxLosses*, the harder learning task of predicting sepsis preferred a stronger integration of priors as *AuxLosses@Branches*. Although selecting the level of prior integration was on average not the most important decision for learning muscle function, the highest positive impact on predictive performance was found with strong prior integration, namely *Branches* and *AuxLosses@Branches*, for active and passive force. The results for pCa50 are harder to interpret. According to the individual SHAP values, the task preferred no prior integration or weak integration as *AuxLosses* but the pattern of the association is rather complex. Further explanation, for instance, why a prior integration *AuxLoss* can be considered weaker than Branches, can be found in Methods.

Most learning tasks, especially *mdx* and sepsis, benefited from smaller NN capacity, indicating that fewer DOF were sufficient for the complexity of the task and helped to avoid overfitting.

Reducing image resolution had a negative impact on five of seven learning tasks, although at varying degrees, as indicated by the SHAP values when employing down‐sampling. Especially *mdx* profited from a higher resolution. Those tasks required detailed information of highly resolved muscle filament structures, while especially sepsis worked better at downsampled resolution, indicating that future imaging data could be recorded at higher throughput for this task.

Prediction of active and passive force benefited from contrast enhancement. This is also intuitively comprehensible when inspecting the images visually, as the IQ for function assessment is lower on average due to a more complex experimental setup^[^ ^31^ ^]^ (Supporting Information S1). On the contrary, the modification of image intensities by contrast enhancement had a negative effect for the tasks *mdx*, muscle type and sepsis. This indicates that not only the structure, but also the original intensity yields important information for these tasks and should not be artificially modified.

In two of seven tasks, the selection of the spacing between three representative slices was the most important decision. Interestingly, for active and passive force prediction, SEMPAI strongly profited from using slices from the periphery of the muscle fiber (±20 µm), compared to using further slices in the proximity of the muscle center (i.e., 1, 5, and 10 µm). This indicates additional biological information for function assessment in the muscle periphery in comparison to a sole evaluation of the muscle center. Using configurations that employed 3D DaRe generally provided an inferior predictive performance, and none of these models was found among the best 100 for any task.

*mdx* was the only task for which localized properties were of special interest, since the performance decreased by employing random erasing.^[^ ^48^ ^]^ We explain this technique, and how we use it, in Methods. In brief, random erasing is an augmentation method that regularizes by preventing a model from using only one or a small number of image regions to learn. Random erasing of a few image regions prevents this, similar to the concept of dropout^[^ ^49^ ^]^ for neurons. However, if an information occurs only locally, random erasing leads to a situation where prediction is no longer possible. To investigate this effect for *mdx*, we used the sample‐level decision explanation of SEMPAI. This confirmed that, in addition to 2D‐VD, localized regions of twisted or damaged muscle fibers were especially used to predict *mdx*. An example of such a finding is shown in (Figure 3B). When those image regions were randomly erased, a loss of predictive performance was observed. For all other tasks, however, more global properties seem to be important for prediction, making the augmentation effect^[^ ^48^ ^]^ of random erasing more advantageous.

As demonstrated for *mdx*, SEMPAI provides a detailed sample‐level highlighting of important image regions, orthogonally to the information given by priors. A collection of examples is shown in Supporting Information S2. However, for a proper quantitative evaluation, those observations must be validated in a standardized manner, which is beyond the scope of this study. In the future, an observer study based on SEMPAI could lead to novel scientific insights.

In most of its decisions, SEMPAI autonomously chose a stronger regularization. This was achieved by a strong prior integration, a low NN capacity, and also the lower dimensional 2.5D DaRe.

### 2.5 Predictive Performance of SEMPAI's Foundation Models and Comparison to Benchmarks

To benchmark the performance of SEMPAI, we implemented two state‐of‐the‐art (SOTA) baselines: as univariate analyses still reflect the standard approach in laboratory research, especially in a low sample size setting, we select the best prior on the combined train and dev set and use it as a univariate predictor for the test set. In addition, to assess the performance of SOTA multivariate modeling, we use all priors and fit a statistical pipeline, consisting of MRMR^[^ ^50^ ^]^ feature selection, best subset selection. and multiple linear/logistic regression, on the combined train and dev set. The resulting model is applied for the prediction on the test set. For fair benchmarking, as statistical models are more severely regularized, potentially resulting in underfitting, we vary the best subset selection information criterion (Akaike/Bayesian) and the penalty of the regression (L2/elastic net: L1&L2) and report the best performance on the test set. To understand the merit of priors and images individually, we report the results of SEMPAI when using only priors (SEMPAI *PriorsOnly*), i.e., when it does not have access to the images, and the opposite, i.e., exclude trials that integrated priors (SEMPAI *NoPriors*). Finally, to test susceptibility of SEMPAI for non‐optimal configurations, we give the average performance of the 50 best models (SEMPAI50).

**Figure 4:**
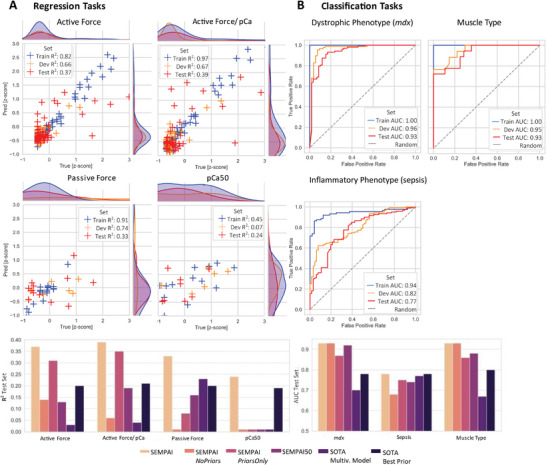
Overall results of SEMPAI, its sub‐configurations and comparison with state‐of‐the‐art (SOTA) methods. Performance metrics in train (for NN training), dev (for meta‐optimization) and test set (unseen data) for regression A), R^2^, and classification, AUC, tasks B).

The detailed results of SEMPAI, including train and dev set performance, and the comparison with SOTA are shown in **Figure** **4** and **Table**  **3**. In six of seven investigated learning tasks, SEMPAI's foundation models were superior to SOTA models in predicting the labels of the test set.

**Table 3:** Overall SEMPAI results in train, dev, and test set; of SEMPAI sub‐configurations, and comparison with SOTA methods (all results on the test set if not denoted otherwise). ns: negative sign, i.e., worse than guessing

Task	SEMPAI Train/Dev/Test	SEMPAI *NoPriors*/ *PriorsOnly*	SEMPAI50	SOTA Multiv. Model	SOTA Best Prior
*Mdx* [AUC]	1.0/0.96/0.93	0.93/0.87	0.92	0.70	*2D‐VD*: 0.78
Sepsis [AUC]	0.94/0.82/0.77	0.68/0.75	0.74	0.77	*3D‐SL*: 0.77
Muscle Type [AUC]	1.0/0.95/0.93	0.93/0.86	0.88	0.67	*2D‐VD*: 0.80
Active Force [R^2^]	0.82/0.66/0.37	0.14/0.31	0.13	0.03	*2D‐CAS*: 0.20
Active Force/pCa [R^2^]	0.97/0.67/0.39	0.06/0.35	0.19	0.04	*3D‐CAS*: 0.21
Passive Force [R^2^]	0.91/0.74/0.33	ns/0.08	0.16	0.23	*2D‐CAS*: 0.20
pCa50 [R^2^]	0.45/0.07/0.24	ns/ns	ns	0.01	*3D‐CAS*: 0.19

Active force was predicted by SEMPAI with *R^2^* 0.37, while SOTA gave 0.20 using the prior 2D‐CAS. The prediction of the biologically more interesting active force adjusted for pCa yielded similar results with a performance of *R^2^* 0.39 by SEMPAI and 0.21 for SOTA by prior 3D‐CAS. For passive force, SEMPAI again achieved solid results with *R^2^* 0.33, while SOTA achieved 0.23 via the multivariate model. For pCa50, SEMPAI was only slightly superior, *R^2^* 0.24, to using the prior 3D‐CAS, *R^2^* 0.19. Predictions of force parameters were more susceptible to performance decrease for non‐optimal configurations than those of pathologies and muscle type, evident from the results for SEMPAI50, which in the case of the force predictions showed inferior results compared to the best trial.

As expected, the prediction for tasks with very small sample size, pCa50 and passive force, was problematic for models with large DOF or without strict regularization as shown by the predictive performance of DL (SEMPAI *NoPriors)*, single‐task AutoML (SEMPAI *PriorsOnly*) and, in case of pCa50, even a simple multivariate statistical model with only few DOF. SEMPAI's regularization by multi‐task learning, integration of priors, and the model selection based on the *total meta‐loss*, however, resulted in a SEMPAI model with slightly improved performance compared to the best SOTA approach, the univariate predictor 3D‐SL (one DOF).

In three of seven tasks, SEMPAI *PriorsOnly* was superior to SEMPAI *NoPriors* and especially achieved competitive performance in classification tasks and for predicting active force. The priors already provided the diagnostic information for classifying the inflammatory phenotype sepsis, since no improvement in predictive performance was observed by additional utilization of DL on images.

In contrast, for the dystrophic phenotype *md*x and the muscle type, SEMPAI *NoPriors* yielded very strong models and, in the case of *mdx*, these predictions were superior to those based solely on priors. Thus, the performance of *PriorsOnly* or *NoPriors* models varied largely between tasks. In all tasks, however, SEMPAI identified a level of prior integration on the dev set that led to a good generalizability, i.e., the best predictive performance for the test set.

Especially for the prediction of muscle function, synergistic effects of combining prior knowledge with DL are observed, as SEMPAI provided strongly improved performance compared to DL without priors or models solely based on priors. These effects may be interpreted as a DL‐based prior (or hypothesis) refinement.

## 3 Discussion

We developed a novel Self‐Enhancing Multi‐Photon Artificial Intelligence (SEMPAI) and applied it on a total of 1,298 single muscle fiber 3D SHG images. SEMPAI targets close interaction with biomedical researchers. On the one hand, SEMPAI integrates, tests, and refines prior knowledge or hypotheses of the domain expert. On the other hand, SEMPAI gives systematic feedback about influencing factors for optimal extraction of biologically relevant information. The researchers can therefore use their domain knowledge as input to the method and receive comprehensible and easy‐to‐interpret feedback as output.

The foundation models generated by SEMPAI were superior to previous state‐of‐the‐art (SOTA) biomarkers in predicting active and passive muscle force, pCa50 for Ca^2+^‐activated isometric force, muscular dystrophy phenotype in the *mdx* mouse as well as murine muscle type. To the best of our knowledge, deep learning (DL) was not yet applied to MPM image databases in single muscle fiber research. For muscle research, DL was for example applied to gene data from DMD patients^[^ ^51^ ^]^ or to perform functional evaluation of DMD on ultrasound images.^[^ ^52^ ^]^ Most often, DL in this context is used on clinical MRI data, e.g., for the identification of MRI biomarkers in smaller cohorts (N = 26),^[^ ^53^ ^]^ for image classification^[^ ^54^ ^]^ or for the analysis of larger clinical cohorts (N = 432).^[^ ^55^ ^]^ However, ultrasound and MRI do not offer sufficient resolution to understand DMD and the *mdx* model at the level of individual muscle fibers. Here, MPM has the unique advantages of label‐free image contrast and sub‐cellular resolution. In support of the microscopic approach, SEMPAI also showed that the prediction of most of the prediction tasks benefit from finer resolution.

Usually, imaging‐based biomarker studies are either purely based on priors, especially if the sample size is low like in many clinical imaging studies, or novel DL architectures. However, meta‐learning on our multi‐study data indicates that a prior integration, by varying degrees, in DL methods almost always yielded the best predictive performance, especially for the prediction of muscle function. Recent research, such as known‐operator learning,^[^ ^17^ ^]^ points in a similar direction and has already shown impressive results by integrating known operators, e.g., subtasks with known analytic solutions in image reconstruction algorithms, into NNs to improve task performance, while preserving the reliability of deterministic methods.^[^ ^17^ ^]^ However, the decision to integrate priors in known operator learning is a design choice made before the experiments are conducted. SEMPAI's approach is agnostic and decides based on the current task if priors are needed. The regularization by weak constraints in the form of auxiliary losses^[^ ^1^ ^]^ is particularly interesting as this variant of regularization, in addition to competitive predictive performance for our data, has the benefit of being able to process samples, in which priors are not available or not reliable due to low IQ. SEMPAI has learned the priors during training and implicitly uses them for inference of those cases even without explicit prior computation. A similar concept of regularization, but for dynamical systems, is applied in physics‐informed neural networks,^[^ ^56^ ^]^ which regularize the learning of systems dynamics by known differential equations. Priors are represented by the differential equations that are incorporated into the NN training by losses that use the deviation between predictions made by the NN and those expected following the equations.

Most studies with DL develop/optimize their neural network (NN) architecture for a fixed data representation (DaRe). SEMPAI, however, uses the simultaneous optimization of the DaRe for biological knowledge discovery. Thereby, we showed that most of the investigated learning tasks, as expected, benefit from a higher image resolution. SEMPAI further showed that the muscle periphery is especially important for the assessment of active and passive force measurements or that the distinctive properties of *mdx* dystrophic phenotype are rather learned locally, i.e., at specific locations of the fiber, than globally, i.e., widespread over the whole fiber. However, prediction of *mdx* by SEMPAI is, to a certain extent, also possible using only global characteristics, which is in concordance with recent literature.^[^ ^57^ ^]^ The information provided by SEMPAI can be used to guide future experiments and to refine microscopy hardware specifically for a pathology, e.g., by maintaining high resolution in the case of *mdx* or by decreasing resolution in the case of sepsis to increase throughput. Compared to SEMPAI, the recent ground‐breaking meta‐learning approach of Isensee et al. to the biomedical image segmentation problem^[^ ^3^ ^]^ is more technically driven by evolving its decision‐making around pre‐processing and network topology. SEMPAI, however, focuses its decision‐making rather on integrating and returning interpretable information regarding prior knowledge and biology.

SEMPAI leverages shared patterns using multi‐task learning. The benefit of jointly learning multiple tasks has been shown previously^[^ ^26^, ^58^ ^]^; since it allows for a more robust prediction performance even in those tasks for which only a few positive samples are available. Otherwise, with just a small number of examples insufficient for training a high‐variance model from scratch, relying on an already established prior would often be the only option for the lab scientist. Notably, joint learning is also interesting for biological reasons, as shown in pan‐cancer research,^[^ ^59^ ^]^ since the highlighting of common patterns between related pathologies might be beneficial in the development of appropriate drugs. In addition to joint learning of multiple tasks by the NN, it was suggested that joint meta‐learning, i.e., simultaneous optimization of NN architecture and configurations over different tasks might be beneficial.^[^ ^12^ ^]^ This is explicitly utilized by SEMPAI as well. One main objective of SEMPAI's multi‐task learning approach is to create foundation models, i.e., models trained on a multitude of similar tasks, that are then only fine‐tuned for a novel task. Foundation models are mostly semi‐supervised due to the lack of labels, (pre‐)trained on a variety of similar tasks and adapted to the respective application by domain adaptation. For this purpose, further experiments with single muscle fibers and in animal models will be added to the existing database, and, by combining priors and DL, robust foundation models will be generated by SEMPAI. Those foundation models can then be fine‐tuned for MPM endoscopy,^[^ ^60^ ^]^ thereby potentially translating from fundamental research to the clinics.

As one limitation of this study, while intended as a general‐purpose tool, SEMPAI was only evaluated for muscle research. In the future, we plan to expand SEMPAI to other organ models, including existing gastroenterological^[^ ^60^, ^61^, ^62^ ^]^ and pneumonological^[^ ^63^ ^]^ MPM databases and respective priors. Further, SEMPAI did not yield a good predictive performance with 3D DL based on the underlying architecture. The phenomenon that DL approaches using lower‐dimensional “multi‐view” data representations are sometimes superior to DL methods working directly on 3D data is well‐known.^[^ ^64^, ^65^ ^]^ In addition, it is also conceivable that SEMPAI recognized that the data amount was not sufficient for a 3D analysis with significantly more degrees of freedom, and hence regularized itself. However, we believe that further conceptual developments for SEMPAI are required for beneficial use of full 3D information. Another drawback comes from the use of meta‐learning. This is very computationally intensive because a large number of models need to be trained. Since we also utilize meta‐learning for knowledge discovery, we cannot prune the training as much by, e.g., hyperband pruning^[^ ^66^ ^]^ or other aggressive pruners. Also, performance estimation strategies from the NAS domain do not seem reliable enough for knowledge discovery. Thus, a single converged run takes between three to four weeks on a system consisting of a Nvidia RTX3090 GPU and an Intel Core‐i9 10850k CPU, see Methods. Compared to NAS optimizations, e.g., on ImageNet, which have been performed by industry with supercomputers and large costs, the computation time is moderate with our approach. However, with a further increase of the configuration space, e.g., by more adjustments of the NN architecture as in NAS, we will have to resort to more powerful hardware in the future.

In conclusion, in this work we present SEMPAI, an AI specific for laboratory and basic research. It uses meta‐learning for knowledge discovery, allows combining the hypothesis‐driven approach of fundamental research with DL, and shows good predictive performance even for small experiments where DL or machine learning in general would not be rationally applicable. It is strongly regularized and prevents overfitting through several external design choices and internal optimization choices. SEMPAI's decision to integrate priors, utilize NN architectures of low capacity, and use low‐dimensional DaRe were internal optimization choices for regularization. Its utilization of multi‐task learning is an external design choice. We tested this approach with a large exclusive dataset of 3D SHG images of single muscle fibers with a multitude of pathologies and functional properties, which result from over a decade of experiments. The meta‐learning on a large database aims to build foundation models for different organs, which could find future application when translating from ex vivo^[^ ^62^ ^]^ to in vivo experiments^[^ ^60^, ^61^ ^]^ or from animal models to humans. Both through the systematic analysis of differences and similarities between experiments and pathologies and the adaptation of the method by meta‐learning, as well as through the continuous expansion of its database, we expect a continuous self‐enhancement of the method.

## 4 Experimental Section

### Selected Studies

*a. A – inflammatory phenotype (sepsis vs. control)*.^[^ ^30^ ^]^ Sepsis was induced by caecal ligation and puncture (CLP) of 24‐week‐old mice, and the *extensor digitorum longus* (EDL) muscle was extracted. Maximum isometric tetanic forces were induced in the native whole muscle via needle electrodes (Aurora Scientific) by averaging three consecutive tetanic stimuli (150 Hz stimulation frequency, 200 ms duration, 0.2 ms pulse width, 2 min rest intervals). Thereafter, the dissected and in paraformaldehyd (PFA) fixed muscle tissue was imaged with a voxel size of 0.2 × 0.2 × 0.5 µm, in a field‐of‐view of 100 × 100 µm with a stack depth of typically 50 µm. Single fiber biomechanics was assessed using the previously described *MyoRobot* system to measure active force and reconstruct the force‐pCa curve. The 3D‐SL and myofiber diameter were derived at the beginning of the experiment.

*b. B1 & B2 – active force @ dystrophic phenotype (mdx vs. WT)*.^[^ ^31^ ^]^ The age of the mice was between 13 and 21 weeks for WT and between 27 and 91 weeks for *mdx*. Single muscle fiber segments were manually dissected from the native EDL muscle and clamped into the *MechaMorph* system for subsequent force measurements and SHG imaging. The fiber was adjusted so that its SL was in the range of 2.2 – 3.1 µm as shown by the *MechaMorph* system. Then, force measurements were performed to assess active force parameters (see above). The maximum activation was measured at a pCa of 4.92 in an undiluted highly activating solution (HA, mM: Hepes 30, Mg(OH)_2_ 6.05, EGTA 30, CaCO_3_ 29, Na_2_ATP 8, Na_2_CP 10, pH 7.2). Specific force, Hill‐fit and pCa50 were determined. SHG imaging was performed in two different scenarios (B1 & B2). In B1, a 3D SHG image stack was recorded at each single force recording. In B2, the fiber was only imaged in the relaxed state (pCa 9). Single fibers were z‐scanned using a 0.5 µm step size and at a voxel‐size of 0.139 × 0.139 × 0.500 µm^3^.

*c. C – passive force @ dystrophic phenotype (mdx vs. WT)*.^[^ ^31^ ^]^ The overall procedure was the same as in the active force measurements described above (see B1&B2). However, in this case the *MechaMorph* system was used to access passive force parameters. At each step of force recording, an SHG 3D image stack of the fiber was recorded before proceeding to the next stretch step.

*d. D – muscle type (EDL vs. SOL) @ dystrophic phenotype (mdx vs WT) in tissue*. The investigated mice were 9 months of age. Whole muscle tissue from EDL and diaphragm was fixed in 4% PFA and transferred in PBS on dry‐ice for transportation. Each muscle was cut longitudinally at the highest cross‐sectional area. Small cryo‐cuts of 10 µm were performed and collected on microscope slides. Each slice was further investigated by SHG microscopy. VD, CAS, and SL were derived. In some cases (N = 222), images were recorded from whole muscle tissues. In these cases, single fibers were digitally cropped from the 3D image stacks and afterwards standardized. Force recordings were not performed here.

### Label‐Free SHG Imaging and Functional Force Measurements

*a. Label‐free SHG imaging*. Label‐free SHG imaging was performed on an inverse multiphoton microscope (TriMScope, LaVision BioTec, Bielefeld, Germany) with a mode‐locked fs‐pulsed Ti:Sa laser (Chameleon Vision II, Coherent, Santa Clara, CA, USA). The laser was tuned to a wavelength of 810 nm, generating the second harmonic generation signal at 405 nm. The laser was focused into the sample by a water immersion objective (LD C‐Apochromat lens – 40x/1.1/UV–vis‐IR/WD 0.62, Carl Zeiss, Jena, Germany), and the generated SHG signal was detected by an ultra‐sensitive photo multiplier tube (PMT) (H 7422–40 LV 5 M, Hamamatsu Photonics) in transmission mode to target the SHG of myosin‐II.

*b. Functional force measurements via the MyoRobot system*.^[^ ^37^, ^38^ ^]^ The MyoRobot was a biomechatronics system for automated assessment of biomechanical active and passive properties as previously described.

*c. Functional force measurements via the MechaMorph system*.^[^ ^31^ ^]^ The *MechaMorph* was a custom‐engineered device for combined structure–force measurements. A small measurement chamber could be inserted onto the microscope stage below the objective. Single muscle fiber segments could be mounted between a force transducer and a software‐controlled voice coil actuator (VCA) that allows the *MechaMorph* to perform subsequent isometric force measurements and structural imaging via SHG microscopy.

### Priors

*a. Cosine angle sum (CAS)*. The CAS quantifies the angular deviation of myofibrillar bundles from the main axis.^[^ ^46^ ^]^ This well‐established parameter was deduced from 2D planes of SHG images by a software algorithm (2D‐CAS).^[^ ^46^, ^67^, ^68^ ^]^ The CAS describes disturbances in muscle myofibrillar architecture that have been shown to correlate with muscle weakness.^[^ ^33^ ^]^ For that an upgraded version for 3D assessment of CAS (3D‐CAS) was developed recently.

*b. Vernier density (VD)*. Y‐shaped deviations from the regular sarcomere pattern in SHG images are referred to as “verniers”. The number of these verniers was then normalized to the fiber area to obtain the VD. Values close to zero represent fibers, where all myofibrils were perfectly in register, while larger values indicate deviations. The VD can either be generated manually or by a custom‐designed software tool.^[^ ^68^ ^]^

*c. Sarcomere length (SL)*. With the software tools for *MechaMorph* and *MyoRobot*, the SL was recorded live.

*d. Smart Cross‐Sectional Area (CSA) computation*. In the current study, a new method for quantifying the CSA of single muscle fibers is reported, which had been developed for a standardized solution of the CSA in all image data sets. First, a binarization of the images was performed by a simple Otsu threshold on the images. An oriented bounding box algorithm^[^ ^69^ ^]^ was applied to the binarized fiber to orient the fiber vertically. The top and bottom 10 slices were excluded from quantification. Then, three algorithms were combined with each other, and an outlier detection was applied to increase the stability of the method.

- Algorithm 1 – exact counting: Since the binarized fiber was now arranged from top to bottom, morphological operations 2D opening and closing were applied to each slice to close holes and obtain a compact segmentation. After application, the number of pixels in each slice was counted and averaged.
- Algorithm 2 – principal component‐based: Instead of morphological operations, a 2D principal component analysis (PCA) of scikit‐learn was applied and the obtained maximum and minimum radii were used to determine the area of an ellipse for each slice. The results were averaged over the slices.
- Algorithm 3 – elliptic envelope‐based: Instead of morphological operations, an elliptic envelope (EE) was calculated with a contamination of 0.2. The area of the EE was calculated for each slice and the results were averaged across slices.

The mean results of two algorithms, which show higher concordance, were used. The averaging and outlier removal compensates for potential weaknesses of the algorithms due to varying IQ. The results agreed well with visual assessment.

*Implementation of cross‐study standardization and data split*: The pipeline was written in Python (v3.7.7). For studies with low SNR, a median filter of size 1 µm was applied. An intensity threshold for the background by Otsu's thresholding was defined. Then, voxels with intensities below this threshold intensity were set to 0 (background). The contrast enhancement algorithm MCLAHE^[^ ^47^ ^]^ was applied with adaptive histogram range. The registration toolbox Elastix^[^ ^70^ ^]^ was used to register the muscle fibers to a reference fiber, which exhibits a canonical structure and perfectly vertical orientation. A rigid multi‐scale Euler registration with 600 iterations was used, automatic scale estimation, center of gravity initialization, 32 bins, 6 scales, and grid‐adaptive step size. The transformation was then also applied to the non‐enhanced fiber. Each standardized fiber was normalized to a sample‐wise standard score. Force measurements were extracted directly from the TDMS curves coming from the instruments, entered the data frame and normalized by the CSA of the associated fiber. The standardization pipeline was highly automated, and the steps were documented by an automatically generated SEMPAI labbook to identify and minimize errors associated with standardization or data management.

For data splitting in train (2/4), dev (1/4) and test (1/4) set, the data were both stratified and grouped. The stratification was needed to had sufficient data with a certain label in all sets. Continuous functional labels were median‐dichotomized into “high” and “low” values, e.g., specific force “high” for stratification. However, those dichotomized labels were only used for stratification and not as a learning task. This stratification also ensures that class distributions were balanced over train, dev, and test set. The labels were grouped according to muscle bundle, single fibers from one bundle were therefore, not split between train, dev and test set, preventing information leakage.

*Implementation of SEMPAI configuration‐space and self‐enhancement*. SEMPAI was implemented in Python (v3.8.1), its NN parts in PyTorch (v1.11, CUDA v11.3). For meta‐learning, the multi‐objective optimization algorithm NSGA‐II^[^ ^29^ ^]^ from the Optuna^[^ ^71^ ^]^ package was leveraged with population size of 50, without mutation probability, with a crossover probability of 0.9, swapping probability of 0.5, and a fixed seed of 42.

The losses of labels and priors were weighed against each other by uncertainty weighing.^[^ ^28^ ^]^ For this purpose, additional learnable parameters were introduced, that weigh the losses against each other. The loss is, therefore, determined by: $L\;=\sum_{i}\left(\frac{L_{L,i}}{\sigma_{L,i}^{2}}+\log\sigma_{L,i}\right)\;+\sum_{j}\left(\frac{L_{P,j}}{\sigma_{P,j}^{2}}+\log\sigma_{P,j}\right)$ with labels i of set L and priors j of set P, and the learnable uncertainties associated with each label σ_*L*,*i*_ and prior σ_*P*,*j*_. For the *2.5D* DaRes, three 2D slices of the 3D images were fed in three channels of a 2D EfficientNet. The center slice of the cropped bounding box was used and two further peripheral slices, whose distance from the center slice was optimized by SEMPAI. For NN with branches, i.e., SEMPAI *Branches* and *AuxLosses&Branches*, a wrapper was built for the respective NN to introduce the priors in the fully connected layers.

For AutoML based on priors, i.e., SEMPAI *PriorsOnly*, the Tree‐based Pipeline Optimization Tool (TPOT)^[^ ^72^ ^]^ was employed. This algorithm combines identification of feature selection and suitable classifiers or regressors with Pareto optimization. 250 generations was used, a population size of 200, and grouping of the fibers. The combined train & dev set was forwarded to TPOT for training, and the internal cross‐validation (CV) was set to two‐fold to have a comparable data split ratio to the other components of SEMPAI. TPOT was restricted to methods with class probability output. The performance metric, e.g., *AUC* or *R* ^2^, of the internal CV was reported to SEMPAI and evaluated as a meta‐loss, i.e., the model selected by SEMPAI can be a prior‐only model based on AutoML.

The *total meta‐loss* was a weighted sum of each label. The labels were weighted as a trade‐off between sample size and importance of task, accordingly we set weights w = [*mdx*: 1.0, sepsis: 1.0, muscle type: 0.5, active force: 1.0, active force/pCa: 1.0, passive force: 1.0, pCa50 = 1.0]. In the trade‐off between exploration and exploitation, multi‐objective optimization algorithms were lending toward exploration as the performance for different tasks must be optimized. Thus, the configuration space was sufficiently sampled although very unpromising regions of configuration space trials were still under‐sampled. Selecting a criterion time for early termination of the trials was not trivial for multi‐objective optimization trials. Therefore, a very non‐conservative criterion was selected. Accordingly, SEMPAI does not compare trials for termination (and save computation time) as in more modern methods like Hyperband pruning.^[^ ^66^ ^]^ The *total meta‐loss* was smoothed by computation of the moving average of the last 10 epochs. A trial was terminated when the *total meta‐loss* did not decrease for 50 subsequent epochs. The early stopping criterion was set active after the initial 75 epochs, resulting in at least 125 epochs performed per trial. The lowest meta‐loss for each respective task was used to select the respective model for the task. For tasks with scarce data (pCa50, passive force), however, the *total meta‐loss* was used for model selection.

SEMPAI offers sample‐level and model‐level decision explanations. For a more detailed explanation of the sample‐level explanation, the example in Figure 3 was used. The sample‐level explanation uses Deep SHAP^[^ ^15^ ^]^. The explanations were adapted to the DaRe and prior integration variants. For the prior integration variants with branches, where the priors were fed as branches into the fully connected layer of the respective NN architecture, the importance of these priors was calculated simultaneously to the importance of the voxels of the image. In Figure 3, it can be seen that SEMPAI uses the twisted image regions and the vernier density to correctly classify this sample as dystrophic. In addition, a model‐level explanation was provided by SEMPAI: to provide more insights about preferable individual configurations for each target label, SHapley Additive exPlanations (SHAP)^[^ ^15^ ^]^ values were computed. For this purpose, a random forest was trained to predict performance metrics of the dev set based on the configuration space. The SHAP Tree Explainer^[^ ^35^ ^]^ was utilized, which was explicitly designed for tree‐based algorithms like the fitted model. The stability of the results by fitting multiple forests with different random initializations was verified and ensemble sizes (i.e., number of trees). Manually inspecting each resulting plot of two representative labels (*mdx* and active force/pCa) gave rise to the same interpretation.

For an application of SEMPAI to other data bases, e.g., other organs, corresponding handcrafted features must be provided as priors, or their computation must be integrated into SEMPAI. Ideally, these were known biomarkers or good hypotheses. A dataframe must be created for train, dev, and test set, containing the labels, the priors, and the paths to the standardized images. Then the meta‐parameter space must be defined for SEMPAI, i.e., which configurations regarding DaRe, prior integration, and NN architecture will be tested and optimized.

### Rationale for Standardization and Configuration Space

*a. Standardization*: Standardization was intended to minimize technical variance, which is usually present in biomarker research.^[^ ^73^ ^]^ This technical variance can even lead to wrong conclusions of an AI system.^[^ ^74^, ^75^ ^]^ To reduce the impact of technical variance, The image was slightly denoised and resampled to uniform isotropic voxel size. Cropping reduces the dimensionality of the images, and DL can focus only on relevant regions. The alignment of fibers via registration helps to minimize the bounding box and can increase the convergence of the learning process, because CNNs, such as the employed EfficientNet, are not rotation invariant.

*b. Configuration space*:

- The benefit of contrast enhancement for visual recognizability of structures was undisputed. However, it was not yet understood if this enhancement adds value for training an AI. Therefore, SEMPAI validates this explicitly and exemplarily for the MCLAHE^[^ ^47^ ^]^ algorithm.
- Random erasing^[^ ^48^ ^]^ regularizes the learning process by enforcing the use of multiple image regions for inference, theoretically resulting in a more robust prediction. Random erasing can enforce regularization since it prevents the model from overfitting specific image regions. Thus, the model needs to use several different image regions and can thus become stable and less prone to overfitting to localized noise or other undesired effects. In the case of localized biologically‐relevant image properties, however, deleting this location naturally leads to a mis‐evaluation of the image and a decrease in predictive performance. We thus use random sampling as a measure for the importance of localized image features.
- Downsampling and multi‐view representations may support learning by minimizing overfitting. It was scientifically interesting to understand the importance of resolution for learning phenotypes and function, since microscopy research targets finer resolution (lower pixel size), often at the expense of reduced throughput. SEMPAI's decision w.r.t. down‐sampling to elaborate how important the image resolution was for a given learning task was interpreted. In analogy, whether to use 3D data directly for learning was evaluated, or to draw representative 2D slices. Whether using lower dimensional DaRe as NN input via downsampling (reduced voxel size) and sub‐sampling (2.5D vs 3D), was tested improves convergence. The benefit of dimensionality reduction in DL was controversial.^[^ ^76^, ^77^ ^]^ Choosing the spacing of the representative slices was also of biological interest. It allows interpretation of where relevant information was located in 3D, i.e., by interpretably sub‐sampling a lower‐dimensional DaRe from a higher dimensional volume. By this, the origin of the biological information can be narrowed down.
- To test the importance of priors, several prior integration methods were used. Besides both extremes, *NoPriors* and the *PriorsOnly*, priors as auxiliary tasks was used, as branches or as a combination of the latter two, to define a scale of prior integration from “weak to strong”. By defining the priors as auxiliary tasks, they were predicted simultaneously to the labels. Thus, the filter kernels of the NN evolve to predict these auxiliary tasks as well. By using these priors as auxiliary tasks, the network can leverage domain knowledge to learn better representations of the data. Since the prior was only indirectly available for learning a label, it as weak prior integration was considered. With the branches approach, the priors were passed on directly to the fully connected layers of the NN, i.e., theoretically, the NN can completely dispense with the additional image information, which was why it as strong prior integration was considered. By adding a prior, i.e., handcrafted feature, branch to the fully connected layer, the network can learn to combine the learned features of the CNN with the priors, which can potentially improve the accuracy and generalization of the model. This approach can be particularly useful in scenarios where the input data was noisy or incomplete, and the handcrafted features can provide additional information to the network. Multi‐branch approaches have also recently been shown to have positive convergence properties^[^ ^78^ ^]^ for learning. The combination of both methods as an even stronger prior integration was defined. Finally, the use of priors with AutoML, i.e., without images and DL, was defined as the “maximum” of the prior integration scale. Such feature‐based ML approaches can occasionally outperform DL.^[^ ^79^ ^]^ In the optimization of SEMPAI, the added value of the priors for the learning process was evaluated. If models with the hypothesis‐driven priors were superior to models without, or if a prior‐only model shows the same performance as the best DL model, the hypothesis that the prior describes the state of the label well can be considered true. The researcher can thus test hypotheses and these were verified by SEMPAI and, in the case of models with DL, also refined. The biological information of the prior knowledge was evaluated.
- Further adaptations: NN‐specific parameters were more technical and less interpretable but need to be adapted to prior integration and DaRe at hand to achieve a global optimum. The NN capacity was adjusted, as it must be adapted to the available amount of data and the complexity of the learning task. Also, further NN properties like batch size, learning rate, momentum, and optimizer must be fine‐tuned. Gradient clipping, i.e., restricting the gradients, had been theoretically shown to accelerate convergence^[^ ^80^ ^]^ and its benefit was evaluated. Also, the sampling of the data can be modified by imbalance sampling. The employed augmentation uses rotations, shifts, and additive noise patterns, which were identified as variations in the data after inspection of the images by domain experts. Thus, this step can also be interpreted as prior knowledge integration. Augmentation introduces invariance toward the applied modifications to the learning process.

### Computation Details

SEMPAI computed 19 days on a workstation equipped with NVMe SSD, Nvidia RTX 3090 GPU and Intel Core‐i9 10850k CPU (10 cores of 3.6 GHz), resulting in a total of 1,500 evaluated trials. To decrease the computational cost for evaluating 2D configurations, the slices were loaded by reading parts of the memory‐mapped 3D volume. For (3D) augmentations, some operations employ TorchIO,^[^ ^81^ ^]^ and where possible, augmentations were computed on the GPU. Automated mixed precision (AMP) of PyTorch was used in addition to multiple workers and pinned memory. To be able to use sufficiently large batches for 3D data, SEMPAI utilizes gradient accumulation.

## Conflict of Interest

The authors declare no conflict of interest.

## Author Contributions

S.N., D.S., C.G., P.R., S.S. conducted the original experiments. L.K., A.M., P.R. curated the data. A.M. designed the research. A.M. conceptualized and developed the method and the software. A.M., L.K. wrote the initial draft; all authors revised the draft. A.M., L.K., P.R., M.H., S.L., D.N., R.H., W.H.G., O.F. conducted the literature review. A.K.M., W.H.G., L.K., O.F. supervised the work. L.K., P.R. evaluated the integrity of multi‐study data, labels, and priors. O.T., S.L., F.D. evaluated the integrity of the method. O.T., F.D., S.L. reviewed the method development and software. D.N, R.H., W.H.G., O.F. contributed to the interpretation of the results.

## Supporting information

Supporting Information

## Data Availability

The complete code is provided on Google Colab under https://colab.research.google.com/drive/18foBUuWKZEVPNuvYaRfmczlaWaaMg6mP All dependencies are pre‐installed and standardized images are provided for rapid execution of the SEMPAI software. The code to standardize the images is also being provided. Finally, the log of a second non‐converged SEMPAI run for another data split is also made available along with analysis scripts, to enable a deeper understanding.

## References

[1] B. S. Doken , T. Zhuang , D. Wingerter , M. Gidwani , N. Mistry , L. Ladic , A. Kamen , M. E. Abazeed , The Lancet Digital Health 2019, 1, e136. 31448366 10.1016/S2589-7500(19)30058-5 PMC6708276

[2] S. M. Humphries , A. M. Notary , J. P. Centeno , M. J. Strand , J. D. Crapo , E. K. Silverman , D. A. Lynch , Radiology 2020, 294, 434. 31793851 10.1148/radiol.2019191022 PMC6996603

[3] F. Isensee , P. F. Jaeger , S. A. A. Kohl , J. Petersen , K. H. Maier‐Hein , Nat. Methods 2021, 18, 203. 33288961 10.1038/s41592-020-01008-z

[4] G. Litjens , T. Kooi , B. E. Bejnordi , A. A. A. Setio , F. Ciompi , M. Ghafoorian , J. A. W. M. Van Der Laak , B. Van Ginneken , C. I. Sánchez , Med. Image Anal. 2017, 42, 60. 28778026 10.1016/j.media.2017.07.005

[5] G. Aresta , T. Araújo , S. Kwok , S. S. Chennamsetty , M. Safwan , V. Alex , B. Marami , M. Prastawa , M. Chan , M. Donovan , G. Fernandez , J. Zeineh , M. Kohl , C. Walz , F. Ludwig , S. Braunewell , M. Baust , Q. D. Vu , M. N. N. To , E. Kim , J. T. Kwak , S. Galal , V. Sanchez‐Freire , N. Brancati , M. Frucci , D. Riccio , Y. Wang , L. Sun , K. Ma , J. Fang , et al., Med. Image Anal. 2019, 56, 122. 31226662 10.1016/j.media.2019.05.010

[6] A. Esteva , B. Kuprel , R. A. Novoa , J. Ko , S. M. Swetter , H. M. Blau , S. Thrun , Nature 2017, 542, 115. 28117445 10.1038/nature21056 PMC8382232

[7] O. Ronneberger , P. Fischer , T. Brox , in International Conference on Medical Image Computing and Computer‐Assisted Intervention, Springer, 2015, 234.

[8] Y. F. Cheng , M. Strachan , Z. Weiss , M. Deb , D. Carone , V. Ganapati , Opt. Express 2019, 27, 644. 30696147 10.1364/OE.27.000644

[9] E. Nehme , L. E. Weiss , T. Michaeli , Y. Shechtman , Optica 2018, 5, 458.

[10] M. Weigert , U. Schmidt , T. Boothe , A. Müller , A. Dibrov , A. Jain , B. Wilhelm , D. Schmidt , C. Broaddus , S. Culley , M. Rocha‐Martins , F. Segovia‐Miranda , C. Norden , R. Henriques , M. Zerial , M. Solimena , J. Rink , P. Tomancak , L. Royer , F. Jug , E. W. Myers , Nat. Methods 2018, 15, 1090. 30478326 10.1038/s41592-018-0216-7

[11] T. Elsken , J. H. Metzen , F. Hutter , J. Mach. Learn. Res. 2019, 20, 1997.

[12] K. O. Stanley , J. Clune , J. Lehman , R. Miikkulainen , Nat. Mach. Intell. 2019, 1, 24.

[13] Y. Liu , W. Zhao , L. Liu , D. Li , S. Tong , C. L. P. Chen , IEEE Transactions on Neural Networks and Learning Systems 2021, 34, 2732. 10.1109/TNNLS.2021.3107600 34520366

[14] S. Jiao , Y. Gao , J. Feng , T. Lei , X. Yuan , Opt. Express 2020, 28, 3717. 32122034 10.1364/OE.382319

[15] S. M. Lundberg , S.‐I. Lee , Adv. Neural Inf. Process Syst. 2017, 30.

[16] C. Rudin , Nat. Mach. Intell. 2019, 1, 206. 35603010 10.1038/s42256-019-0048-x PMC9122117

[17] A. K. Maier , C. Syben , B. Stimpel , T. Würfl , M. Hoffmann , F. Schebesch , W. Fu , L. Mill , L. Kling , S. Christiansen , Nat. Mach. Intell. 2019, 1, 373. 31406960 10.1038/s42256-019-0077-5 PMC6690833

[18] L. Tian , J. Wang , L. Waller , Opt. Lett. 2014, 39, 1326. 24690738 10.1364/OL.39.001326

[19] R. Horstmeyer , C. Yang , Opt. Express 2014, 22, 338. 24514995 10.1364/OE.22.000338 PMC3926543

[20] M. E. Kandel , Y. R. He , Y. J. Lee , T. H.‐Y. Chen , K. M. Sullivan , O. Aydin , M. T. A. Saif , H. Kong , N. Sobh , G. Popescu , Nat. Comm. 2020, 11, 6256. 10.1038/s41467-020-20062-x PMC7721808 33288761

[21] C. L. Cooke , et al., Proceedings of the IEEE/CVF International Conference on Computer Vision 2021, 3803.

[22] M. Moor , O. Banerjee , Z. S. H. Abad , H. M. Krumholz , J. Leskovec , E. J. Topol , P. Rajpurkar , Nature 2023, 616, 259. 37045921 10.1038/s41586-023-05881-4

[23] R. Bommasani , et al., arXiv preprint arXiv:2108.07258, 2021.

[24] L. Parmentier , O. Nicol , L. Jourdan , M.‐E. Kessaci , in 2019 IEEE 31st International Conference on Tools with Artificial Intelligence (ICTAI), IEEE, 2019, 471.

[25] M. Tan , Q. Le , in International Conference on Machine Learning, PMLR, 2019, 6105.

[26] R. M. Caruana , Multitask Learning 1997, 28, 41.

[27] G. M. Van De Ven , H. T. Siegelmann , A S. Tolias , Nat. Comm. 2020, 11, 4069. 10.1038/s41467-020-17866-2 PMC7426273 32792531

[28] A. Kendall , Y. Gal , R. Cipolla , in Proceedings of the IEEE Conference on Computer Vision and Pattern Recognition 2018, 7482.

[29] K. Deb , A. Pratap , S. Agarwal , T. Meyarivan , IEEE Trans. Evol. Comput. 2002, 6, 182.

[30] C. Goossens , R. Weckx , S. Derde , L. Van Helleputte , D. Schneidereit , M. Haug , B. Reischl , O. Friedrich , L. Van Den Bosch , G. Van Den Berghe , L. Langouche , J. Cachexia Sarcopeni. 2021, 12, 443. 10.1002/jcsm.12668 PMC8061378 33465304

[31] D. Schneidereit , S. Nübler , G. Prölß , B. Reischl , S. Schürmann , O. J. Müller , O. Friedrich , Light Sci. Appl. 2018, 7, 79. 30374401 10.1038/s41377-018-0080-3 PMC6199289

[32] P. Ritter , International journal of molecular sciences 2022, 23, 10841. 35708348 10.3390/ijms23126606 PMC9202361

[33] S. Diermeier , et al., Stem Cells Int 2017, 7.

[34] S. Diermeier , M. Haug , B. Reischl , A. Buttgereit , S. Schürmann , M. Spörrer , W. H. Goldmann , B. Fabry , F. Elhimine , R. Stehle , G. Pfitzer , L. Winter , C. Clemen , R. Schröder , O. Friedrich , Biophys. J. 2016, 110, 303a.

[35] S. M. Lundberg , G. Erion , H. Chen , A. Degrave , J. M. Prutkin , B. Nair , R. Katz , J. Himmelfarb , N. Bansal , S.‐I. Lee , Nat. Mach. Intell. 2020, 2, 56. 32607472 10.1038/s42256-019-0138-9 PMC7326367

[36] A. E. H. Emery , Clin. Genet. 1983, 23, 198.

[37] M. Haug , C. Meyer , B. Reischl , G. Prölß , S. Nübler , S. Schürmann , D. Schneidereit , M. Heckel , T. Pöschel , S. J. Rupitsch , O. Friedrich , Biosens. Bioelectron. 2019, 138, 111284. 31103932 10.1016/j.bios.2019.04.052

[38] M. Haug , B. Reischl , G. Prölß , C. Pollmann , T. Buckert , C. Keidel , S. Schürmann , M. Hock , S. Rupitsch , M. Heckel , T. Pöschel , T. Scheibel , C. Haynl , L. Kiriaev , S. Head , O. Friedrich , Biosens. Bioelectron. 2018, 102, 589. 29245144 10.1016/j.bios.2017.12.003

[39] O. Friedrich , M. Haug , B. Reischl , G. Prölß , L. Kiriaev , S. I. Head , M. B. Reid , The International Journal of Biochemistry, Cell Biology 2019, 114, 105563. 31255723 10.1016/j.biocel.2019.105563

[40] D. G. Moisescu , Nature 1976, 262, 610. 958428 10.1038/262610a0

[41] A. Fischmann , P. Hafner , M. Gloor , M. Schmid , A. Klein , U. Pohlman , T. Waltz , R. Gonzalez , T. Haas , O. Bieri , D. Fischer , J. Neurol. 2013, 260, 969. 23138982 10.1007/s00415-012-6733-x

[42] M. Liu , N. Chino , T. Ishihara , Arch. Phys. Med. Rehabil. 1993, 74, 507. 8489361 10.1016/0003-9993(93)90115-q

[43] M. Jansen , N. Van Alfen , M. W. G. Nijhuis Van Der Sanden , J. P. Van Dijk , S. Pillen , I. J. M. De Groot , Neuromuscular Disord. 2012, 22, 306. 10.1016/j.nmd.2011.10.020 22133654

[44] N. Brouilly , C. Lecroisey , E. Martin , L. Pierson , M.‐C. Mariol , H. Qadota , M. Labouesse , N. Streichenberger , N. Mounier , K. Gieseler , Hum. Mol. Genet. 2015, 24, 6428. 26358775 10.1093/hmg/ddv353

[45] M. Dubreuil , F. Tissier , L. Le Roy , J.‐P. Pennec , S. Rivet , M.‐A. Giroux‐Metges , Y. Le Grand , Biomed. Opt. Express 2018, 9, 6350. 31065433 10.1364/BOE.9.006350 PMC6490978

[46] C. S. Garbe , A. Buttgereit , S. Schurmann , O. Friedrich , IEEE Trans. Biomed. Eng. 2012, 59, 39. 21908249 10.1109/TBME.2011.2167325

[47] V. Stimper , S. Bauer , R. Ernstorfer , B. Scholkopf , R. P. Xian , IEEE Access 2019, 7, 165437.

[48] Z. Zhong , L. Zheng , G. Kang , S. Li , Y. Yang , in Proceedings of the AAAI Conference on Artificial Intelligence, 2020, 34, 13001. 10.1609/aaai.v34i04.6175 PMC7710151 33274122

[49] G. E. Hinton , N. Srivastava , A. Krizhevsky , I. Sutskever , R. R. Salakhutdinov , arXiv preprint arXiv:1207.0580, 2012.

[50] C. Ding , H. Peng , J. Bioinf. Comput. Biol. 2005, 3, 185. 10.1142/s0219720005001004 15852500

[51] A. Khamparia , A. Singh , D. Anand , D. Gupta , A. Khanna , N. Arun Kumar , J. Tan , Neural Comput. Appl. 2020, 32, 11083.

[52] A. H. Liao , J.‐R. Chen , S.‐H. Liu , C.‐H. Lu , C.‐W. Lin , J.‐Y. Shieh , W.‐C. Weng , P.‐H. Tsui , Diagnostics 2021, 11, 963. 34071811 10.3390/diagnostics11060963 PMC8228495

[53] A. Mankodi , W. Kovacs , G. Norato , N. Hsieh , W. P. Bandettini , C. A. Bishop , H. Shimellis , R. D. Newbould , E. Kim , K. H. Fischbeck , A. E. Arai , J. Yao , Ann. Clin. Transl. Neurol. 2017, 4, 655. 28904987 10.1002/acn3.440 PMC5590523

[54] J. Cai , F. Xing , A. Batra , F. Liu , G. A. Walter , K. Vandenborne , L. Yang , Pattern Recogn. 2019, 86, 368. 10.1016/j.patcog.2018.08.012 PMC6521874 31105339

[55] M. Yang , et al., BMC Neurol. 2021, 21, 1. 33430797 10.1186/s12883-020-02036-0 PMC7798322

[56] G. E. Karniadakis , I. G. Kevrekidis , L. Lu , P. Perdikaris , S. Wang , L. Yang , Nat. Rev. Phys. 2021, 3, 422.

[57] L. Kiriaev , S. Kueh , J. W. Morley , P. J. Houweling , S. Chan , K. N. North , S. I. Head , Am. J. Physiol.: Cell Physiol. 2021, 321, C704. 34432537 10.1152/ajpcell.00122.2021

[58] E. Meyerson , R. Miikkulainen , in International Conference on Machine Learning, PMLR, 2018, 3511.

[59] J. N. Weinstein , E. A. Collisson , G. B. Mills , K. R. M. Shaw , B. A. Ozenberger , K. Ellrott , I. Shmulevich , C. Sander , J. M. Stuart , Nat. Genet. 2013, 45, 1113. 24071849 10.1038/ng.2764 PMC3919969

[60] A. Dilipkumar , A. Al‐Shemmary , L. Kreiß , K. Cvecek , B. Carlé , F. Knieling , J. Gonzales Menezes , O.‐M. Thoma , M. Schmidt , M. F. Neurath , M. Waldner , O. Friedrich , S. Schürmann , Adv. Sci. 2019, 6, 1801735. 10.1002/advs.201801735 PMC6468963 31016109

[61] L. Kreiß , O.‐M. Thoma , A. Dilipkumar , B. Carlé , P. Longequeue , T. Kunert , T. Rath , K. Hildner , C. Neufert , M. Vieth , M. F. Neurath , O. Friedrich , S. Schürmann , M. J. Waldner , Gastroenterology 2020, 159, 832. 32544392 10.1053/j.gastro.2020.05.081

[62] L. Kreiss , O.‐M. Thoma , S. Lemire , K. Lechner , B. Carlé , A. Dilipkumar , T. Kunert , K. Scheibe , C. Heichler , A.‐L. Merten , B. Weigmann , C. Neufert , K. Hildner , M. Vieth , M. F. Neurath , O. Friedrich , S. Schürmann , M. J. Waldner , Inflamm. Bowel Dis. 2022, 28, 1637. 35699622 10.1093/ibd/izac114 PMC9629455

[63] L. Kreiss , I. Ganzleben , A. Mühlberg , P. Ritter , D. Schneidereit , C. Becker , M. F. Neurath , O. Friedrich , S. Schürmann , M. Waldner , J. Biophotonics 2022, 15, 202200073. 10.1002/jbio.202200073 35611635

[64] E. Ahmed , et al., arXiv Preprint, 1808.01462, 2018.

[65] F. Denzinger , et al., International Conference on Medical Image Computing and Computer‐Assisted Intervention, Springer, 2020, 45.

[66] L. Li , K. Jamieson , G. DeSalvo , A. Rostamizadeh , A. Talwalkar , J. Mach. Lear. Res. 2017, 18, 6765.

[67] O. Friedrich , M. Both , C. Weber , S. Schürmann , M. D. H. Teichmann , F. Von Wegner , R. H. A. Fink , M. Vogel , J. S. Chamberlain , C. Garbe , Biophys. J. 2010, 98, 606. 20159157 10.1016/j.bpj.2009.11.005 PMC2820646

[68] A. Buttgereit , C. Weber , C. S. Garbe , O. Friedrich , J. Pathol. 2013, 229, 477. 23132094 10.1002/path.4136

[69] S. A. Gottschalk , Collision queries using oriented bounding boxes, The University of North Carolina at Chapel Hill, 2000.

[70] S. Klein , M. Staring , K. Murphy , M. A. Viergever , J. Pluim , IEEE Transactions on Medical Imaging 2009, 29, 196. 19923044 10.1109/TMI.2009.2035616

[71] T. Akiba , S. Sano , T. Yanase , T. Ohta , M. Koyama , in Proceedings of the 25th ACM SIGKDD International Conference on Knowledge Discovery, Data Mining 2019, 2623.

[72] R. S. Olson , J. H. Moore , in Automated Machine Learning, Springer, 2019, 151.

[73] J. T. Leek , R. B. Scharpf , H. C. Bravo , D. Simcha , B. Langmead , W. E. Johnson , D. Geman , K. Baggerly , R. A. Irizarry , Nat. Rev. Genet. 2010, 11, 733. 20838408 10.1038/nrg2825 PMC3880143

[74] S. Lapuschkin , S. Wäldchen , A. Binder , G. Montavon , W. Samek , K.‐R. Müller , Nat. Comm. 2019, 10, 1096. 10.1038/s41467-019-08987-4 PMC6411769 30858366

[75] S. Langer , O. Taubmann , F. Denzinger , A. Maier , A. Mühlberg , Bildverarbeitung für die Medizin, Springer Vieweg, Wiesbaden 2023.

[76] T. Poggio , H. Mhaskar , L. Rosasco , B. Miranda , Q. Liao , Int. J. Autom. Comput. 2017, 14, 503.

[77] P. Pope , C. Zhu , A. Abdelkader , M. Goldblum , T. Goldstein , ArXiv Preprint, 2104.08894, 2021.

[78] H. Zhang , J. Shao , R. Salakhutdinov , in The 22nd International Conference on Artificial Intelligence and Statistics, PMLR, 2019, pp. 1099‐1109.

[79] A. Mühlberg , R. Kärgel , A. Katzmann , F. Durlak , P. E. Allard , J. B. Faivre , M. Sühling , M. Rémy‐Jardin , O. Taubmann , Medical physics 2021, 48, 5179. 34129688 10.1002/mp.15049

[80] J. Zhang , T. He , S. Sra , A. Jadbabaie , ArXiv Preprint, 1905.11881, 2019.

[81] F. Pérez‐García , R. Sparks , S. Ourselin , Computer Methods and Programs in Biomedicine 2021, 208, 106236. 34311413 10.1016/j.cmpb.2021.106236 PMC8542803

