# Supplementary material for: SEMPAI: a Self‐Enhancing Multi‐Photon Artificial Intelligence for Prior‐Informed Assessment of Muscle Function and Pathology
Source: Adv Sci (Weinh). 2023 Aug 15;10(28):2206319. doi: 10.1002/advs.202206319 (PMC10558688; doi:10.1002/advs.202206319)
Supplement: Supplementary file 1 — Supporting Information [file ADVS-10-2206319-s001.pdf]

ADVANCED SCIENCE
Open Access

Supporting Information

for *Adv. Sci.*, DOI 10.1002/advs.202206319

SEMPAI: a Self-Enhancing Multi-Photon Artificial Intelligence for Prior-Informed Assessment of Muscle Function and Pathology

Alexander Mühlberg, Paul Ritter, Simon Langer, Chloë Goossens, Stefanie Nübler, Dominik Schneidereit, Oliver Taubmann, Felix Denzinger, Dominik Nörenberg, Michael Haug, Sebastian Schürmann, Roarke Horstmeyer, Andreas K. Maier, Wolfgang H. Goldmann, Oliver Friedrich and Lucas Kreiss*

Supporting Information

SEMPAI: a Self-Enhancing Multi-Photon Artificial Intelligence for prior-informed assessment of muscle function and pathology

Alexander Mühlberg*, Paul Ritter, Simon Langer, Chloë Goossens, Stefanie Nübler, Dominik Schneidereit, Oliver Taubmann, Felix Denzinger, Dominik Nörenberg, Michael Haug, Sebastian Schürmann, Roarke Horstmeyer, Andreas K. Maier, Wolfgang H. Goldmann, Oliver Friedrich, Lucas Kreiss

Supporting Information 1: High-resolution examples for IQ of MPM images after cross-study standardization.

The data is accessible at: https://sempai-mbt.github.io/

1 Study A – SEPSIS
Title/Caption: High-resolution PDF of exemplary fibers of Study A (Sepsis) after standardization without contrast-enhancement.
2 Study A – CE SEPSIS
Title/Caption: High-resolution PDF of exemplary fibers of Study A (Sepsis) after standardization with contrast-enhancement.
3 Study B1 – MDX & ACTIVEFORCE
Title/Caption: High-resolution PDF of exemplary fibers of Study B1 (mdx, active force) after standardization without contrast-enhancement.
4 Study B1 – CE MDX & ACTIVEFORCE
Title/Caption: High-resolution PDF of exemplary fibers of Study B1 (mdx, active force) after standardization with contrast-enhancement.
5 Study B2 – MDX & PCA50
Title/Caption: High-resolution PDF of exemplary fibers of Study B2 (mdx, pCa50) after standardization without contrast-enhancement.
6 Study B2 – CE MDX & PCA50
Title/Caption: High-resolution PDF of exemplary fibers of Study B2 (mdx, pCa50) after standardization with contrast-enhancement.
7 Study C – MDX & PASSIVEFORCE
Title/Caption: High-resolution PDF of exemplary fibers of Study C (mdx, passive force) after standardization without contrast-enhancement.
8 Study C – CE MDX & PASSIVEFORCE
Title/Caption: High-resolution PDF of exemplary fibers of Study C (mdx, passive force) after standardization with contrast-enhancement.
9 Study D – MDX & MUSCLETYPE
Title/Caption: High-resolution PDF of exemplary fibers of Study D (mdx, muscle type) after standardization without contrast-enhancement.
10 Study D – CE MDX & MUSCLETYPE
Title/Caption: High-resolution PDF of exemplary fibers of Study D (mdx, muscle type) after standardization with contrast-enhancement.

Supporting Information 2: Examples for sample-level explanations for image and priors

Configuration of trial with lowest total meta-loss

SEMPAI.config = {'augmentation': True,
'batch size': 'small',
'clipping': True,
'complexity': 2,
'cyclic learning rate': False,
'dimension': '2D_5',
'downsampling': True,
'enhancement': True,
'imbalanced dataset sampling': False,
'lr': 0.0540359659501924,
'momentum': 0.9134831480715462,
'optimizer': 'SGD',
'prior': "aux_loss&branches",
'random erasing': True}

Task *mdx*: Example #1

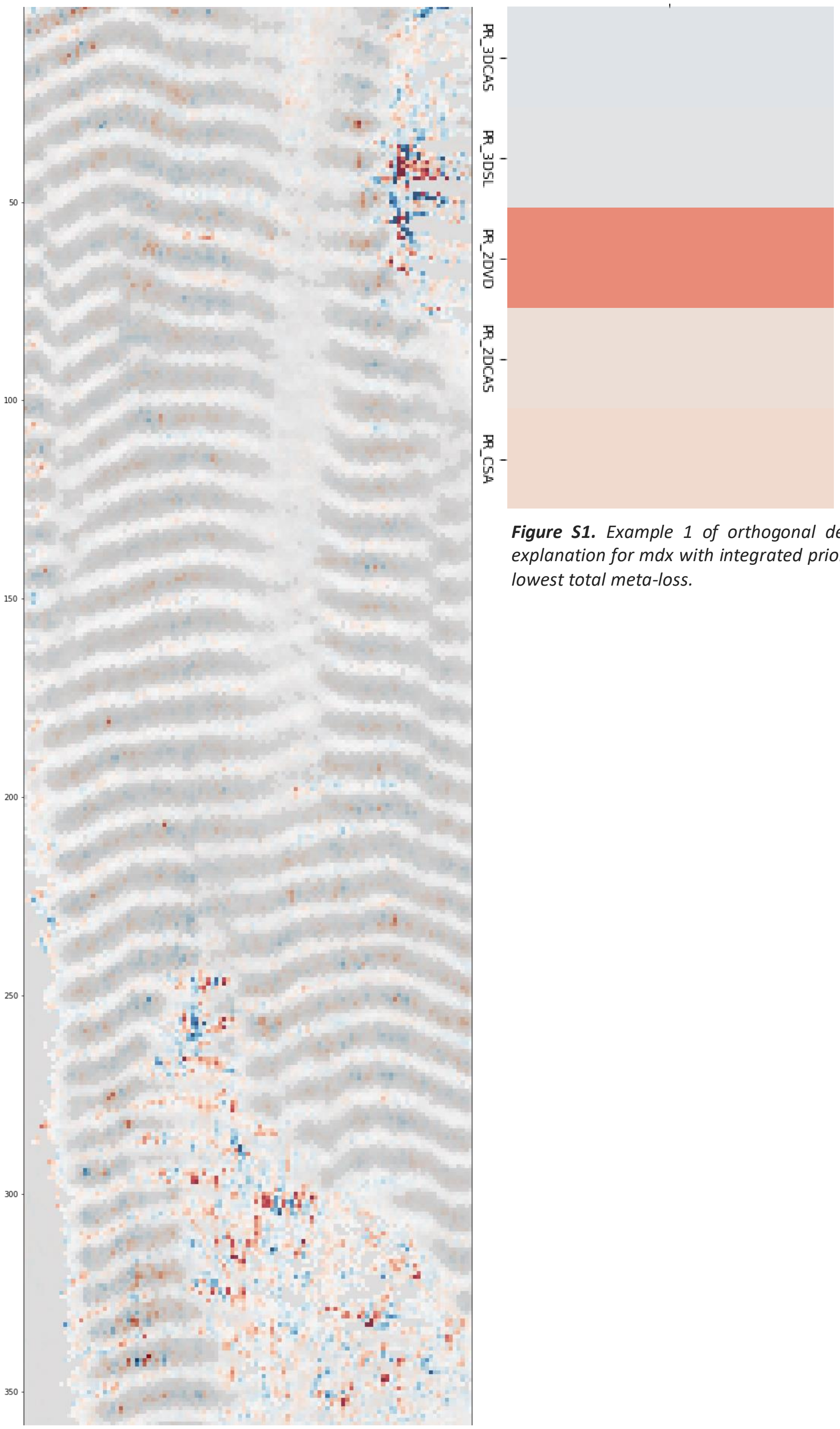

Figure S1. *Example 1 of orthogonal decision explanation for mdx with integrated priors and lowest total meta-loss.*

Example #2

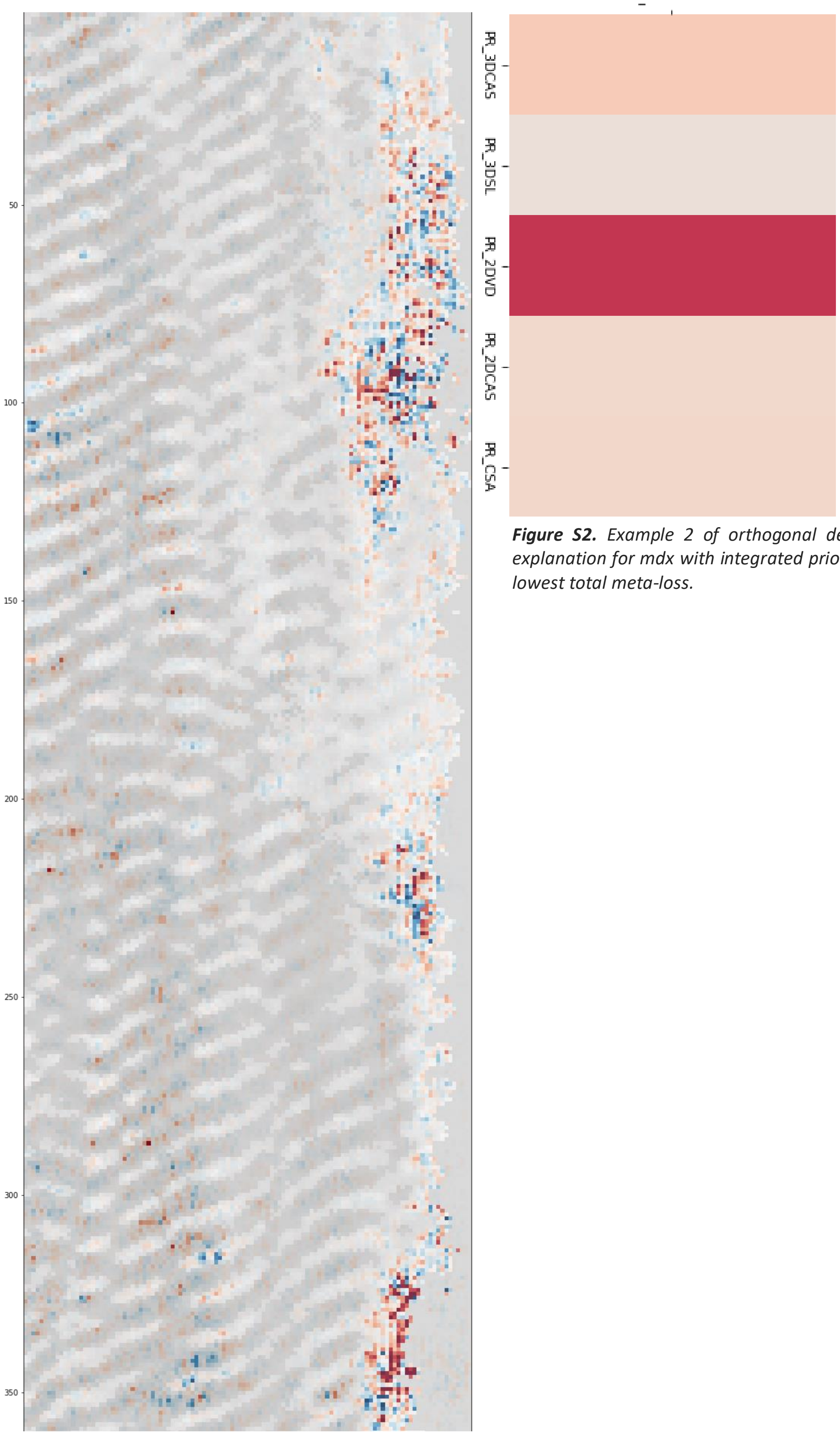

Figure S2. *Example 2 of orthogonal decision explanation for mdx with integrated priors and lowest total meta-loss.*

Task Sepsis: Example #1

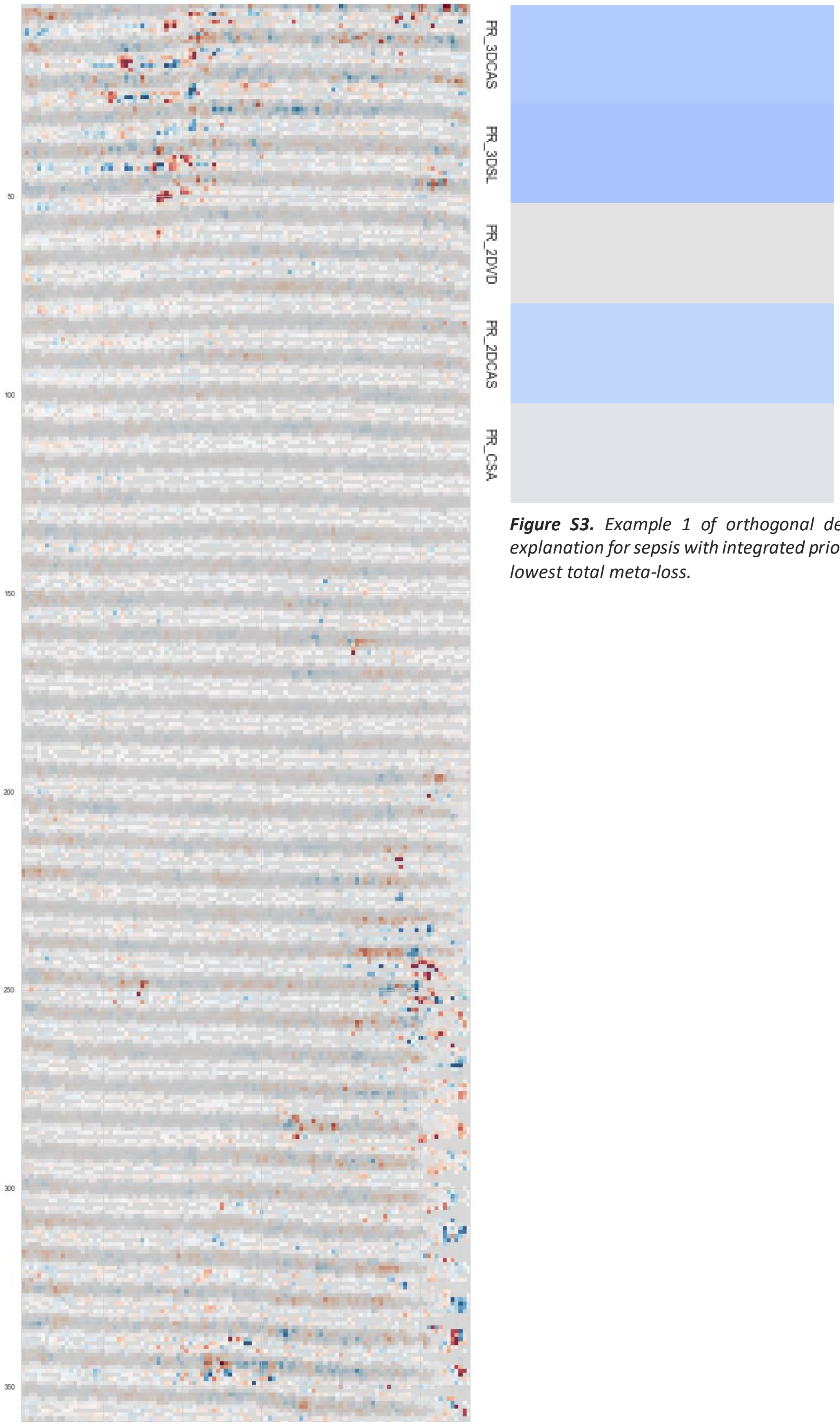

Figure S3. *Example 1 of orthogonal decision explanation for sepsis with integrated priors and lowest total meta-loss.*

Example #2

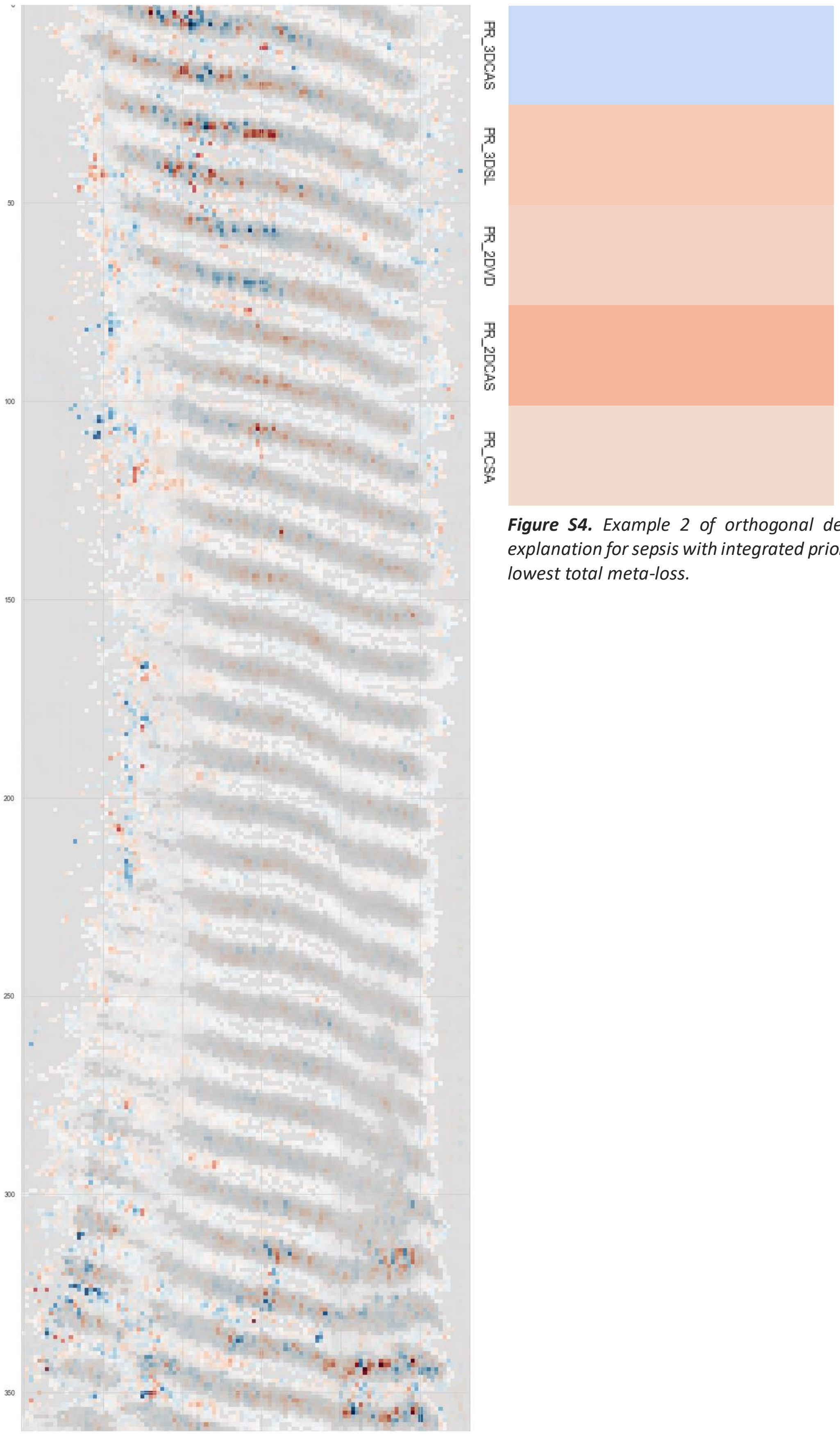

Figure S4. *Example 2 of orthogonal decision explanation for sepsis with integrated priors and lowest total meta-loss.*

Task Muscle Type: Example #1

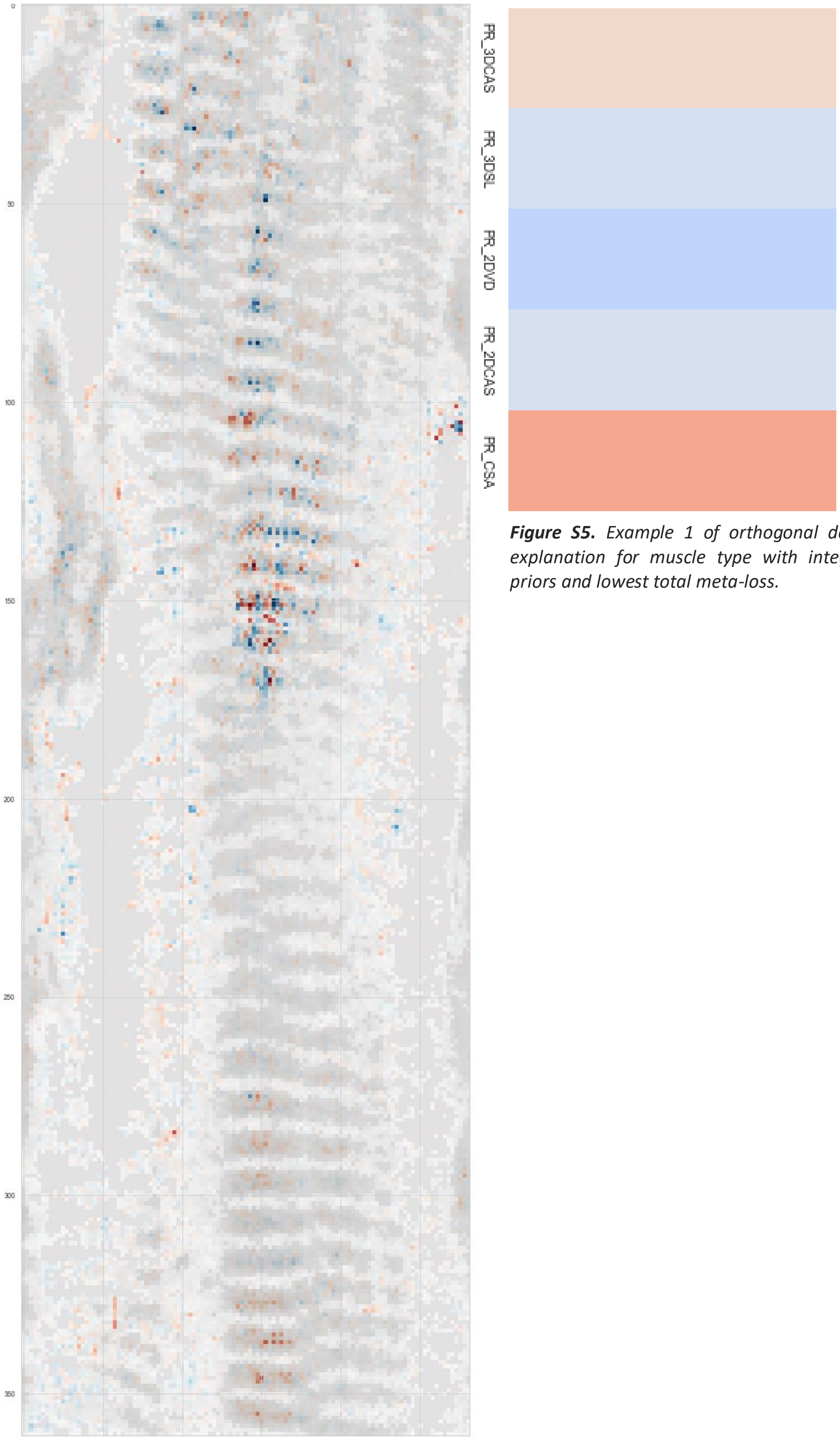

Figure S5. *Example 1 of orthogonal decision explanation for muscle type with integrated priors and lowest total meta-loss.*

Example #2

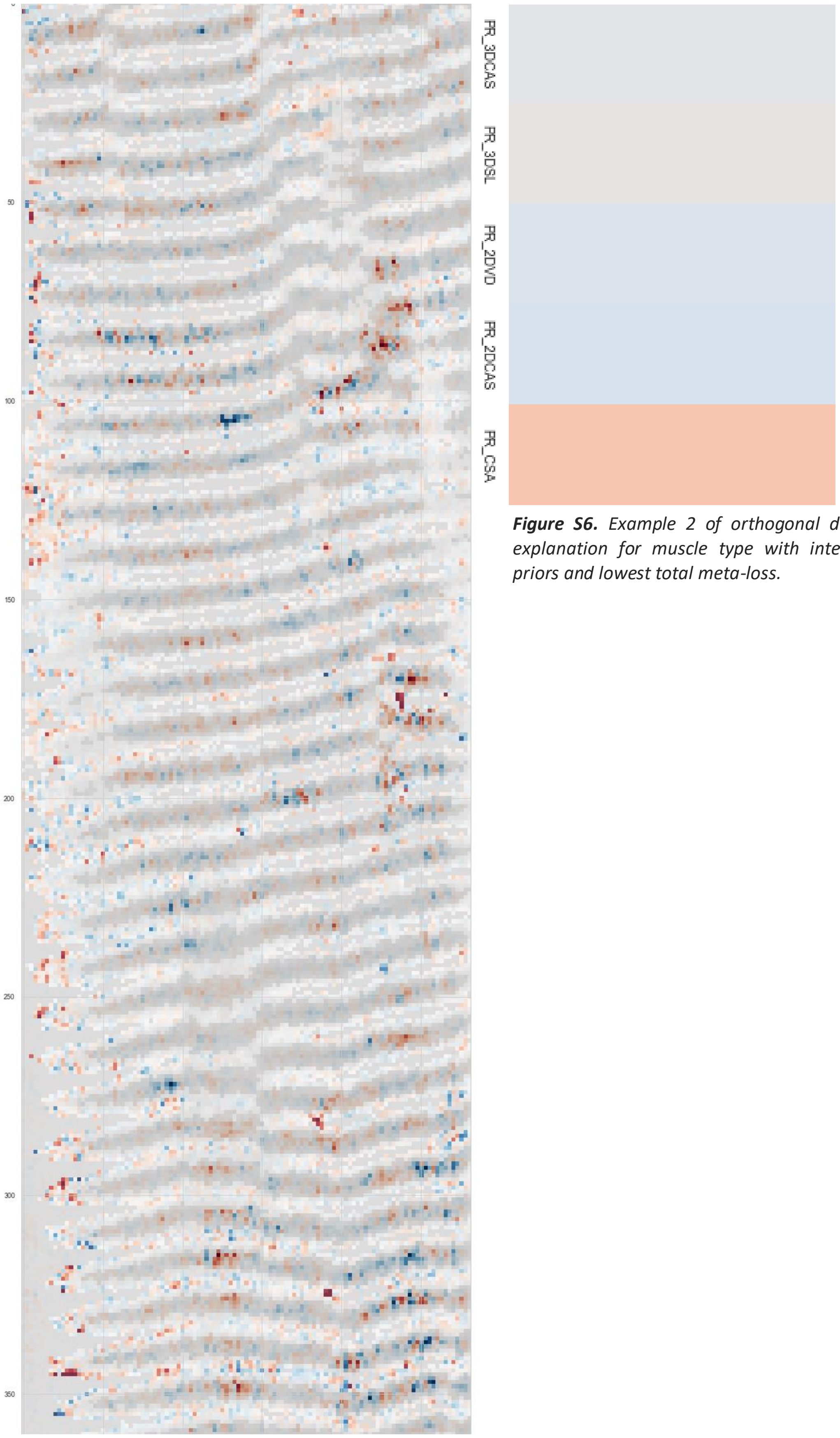

Figure S6. *Example 2 of orthogonal decision explanation for muscle type with integrated priors and lowest total meta-loss.*

Task Active Force: Example #1

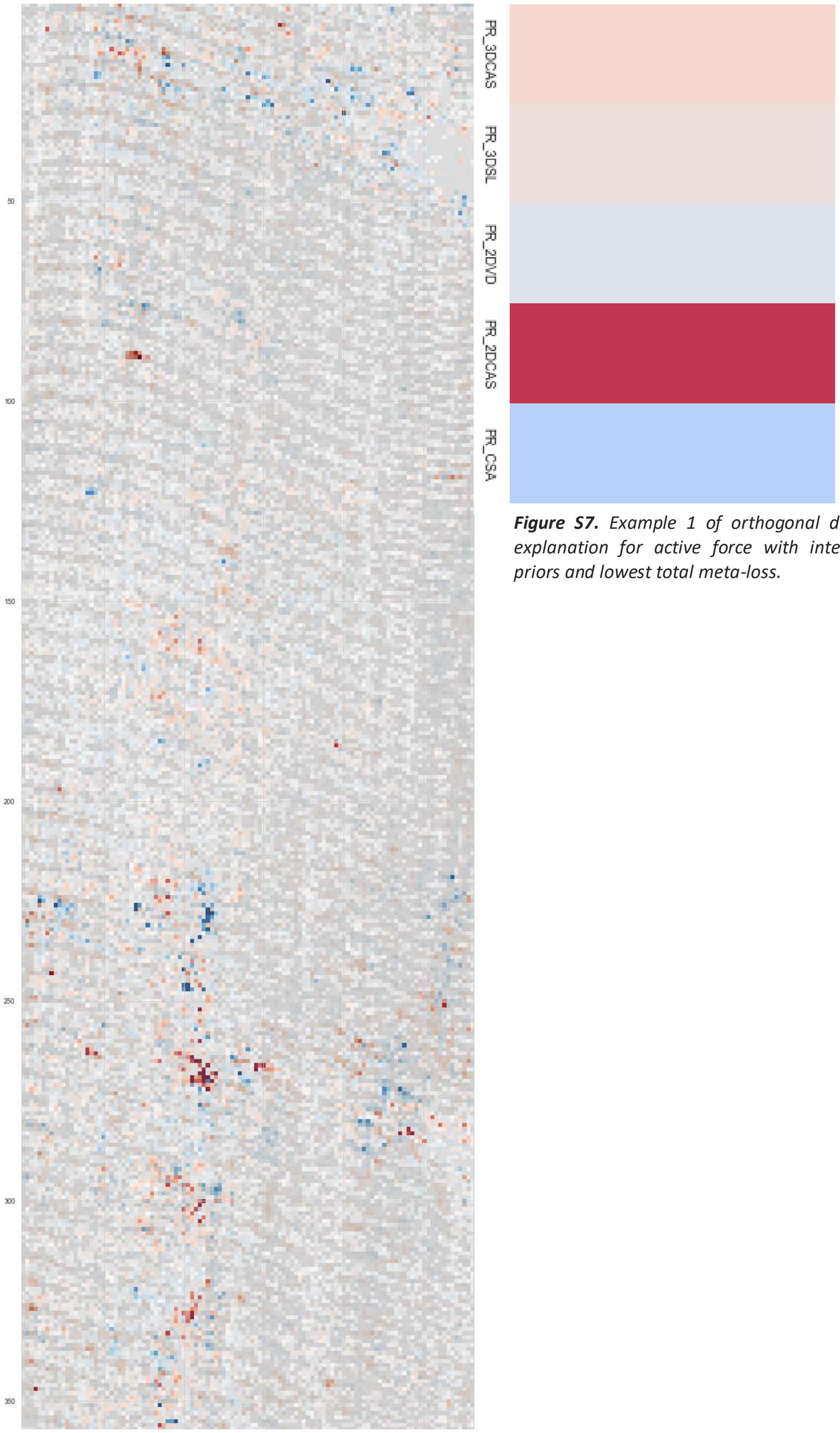

Figure S7. *Example 1 of orthogonal decision explanation for active force with integrated priors and lowest total meta-loss.*

Example #2

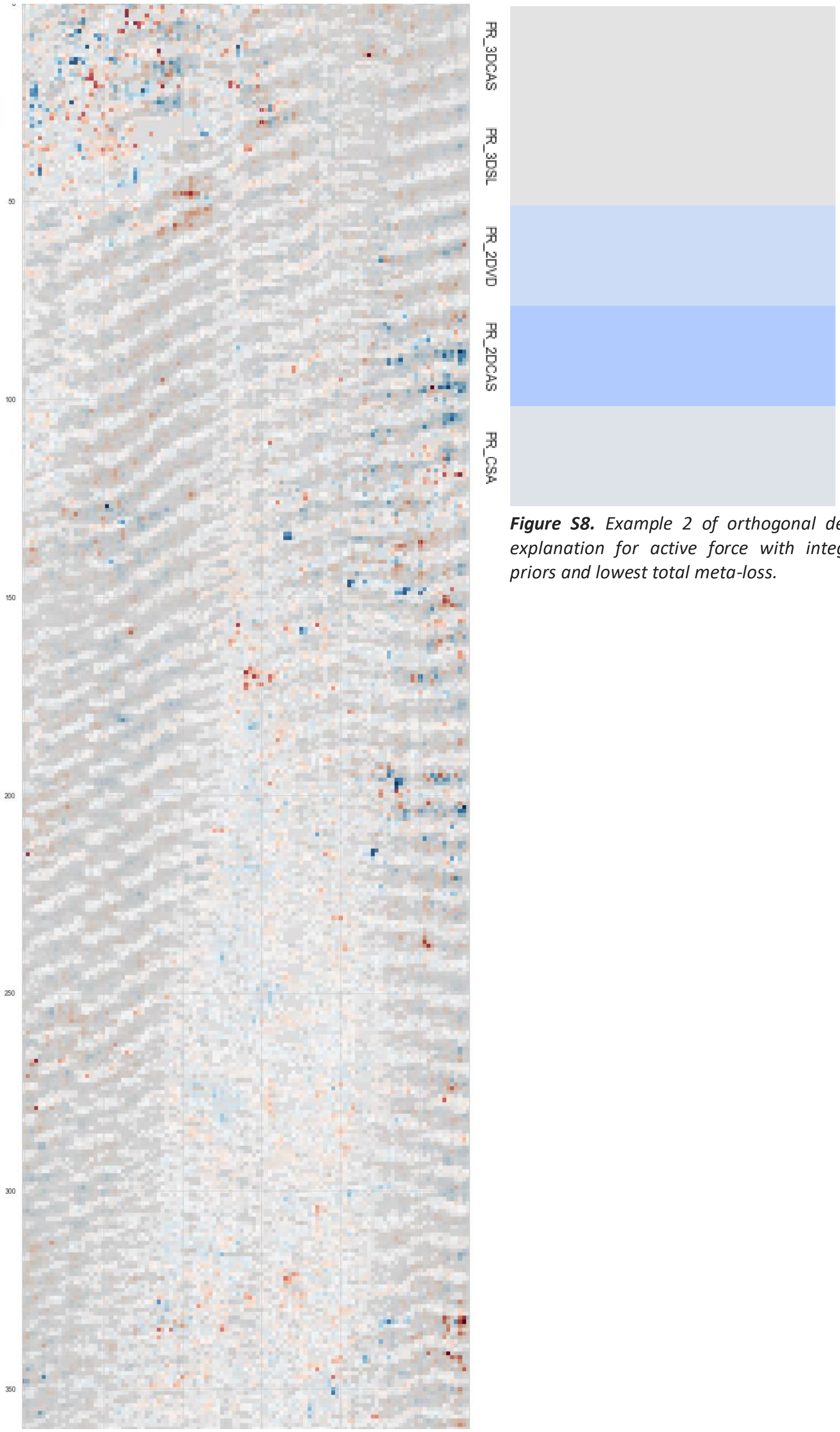

Figure S8. *Example 2 of orthogonal decision explanation for active force with integrated priors and lowest total meta-loss.*

Task Active Force/pCa: Example #1

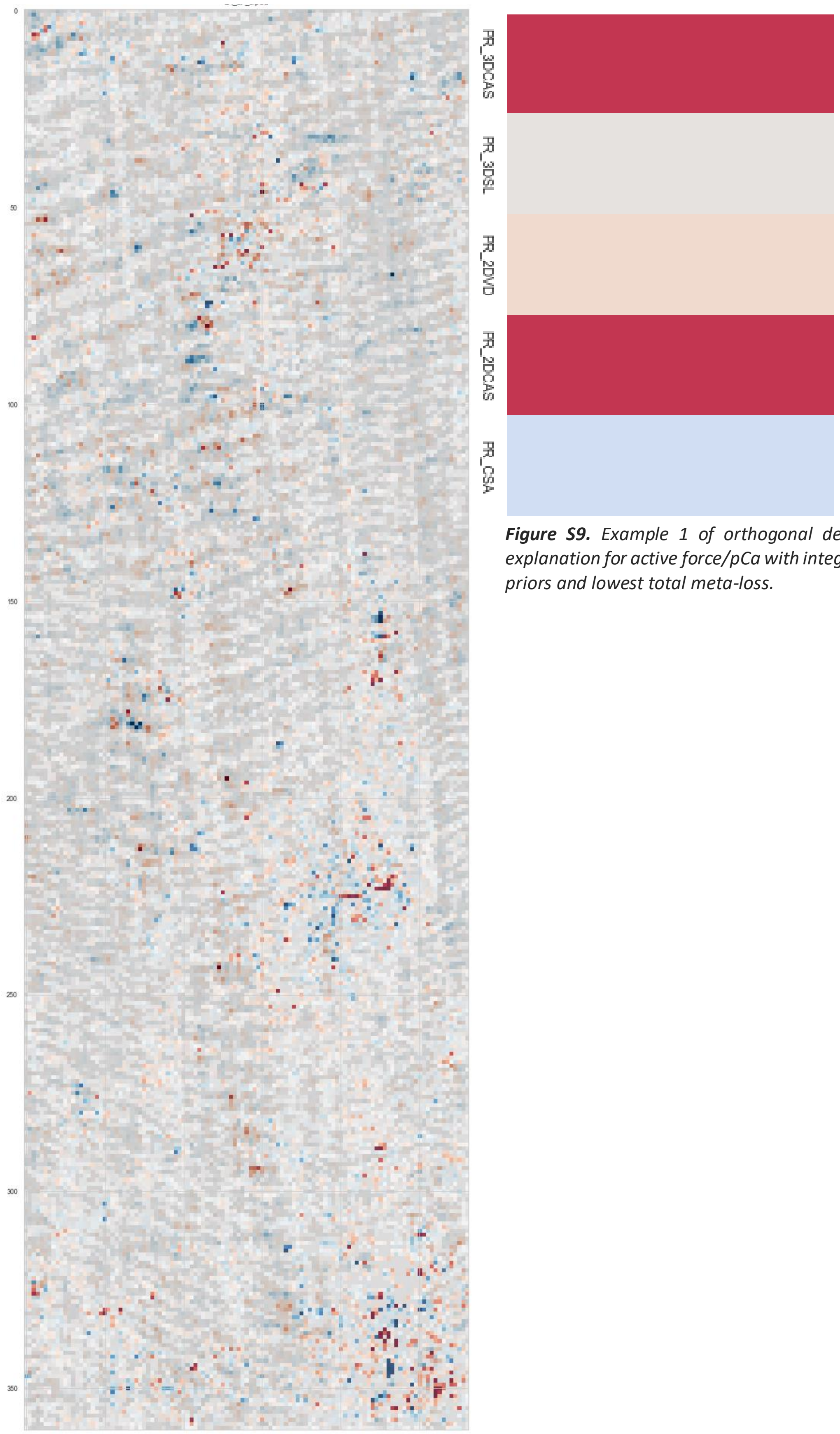

Figure S9. *Example 1 of orthogonal decision explanation for active force/pCa with integrated priors and lowest total meta-loss.*

Example #2

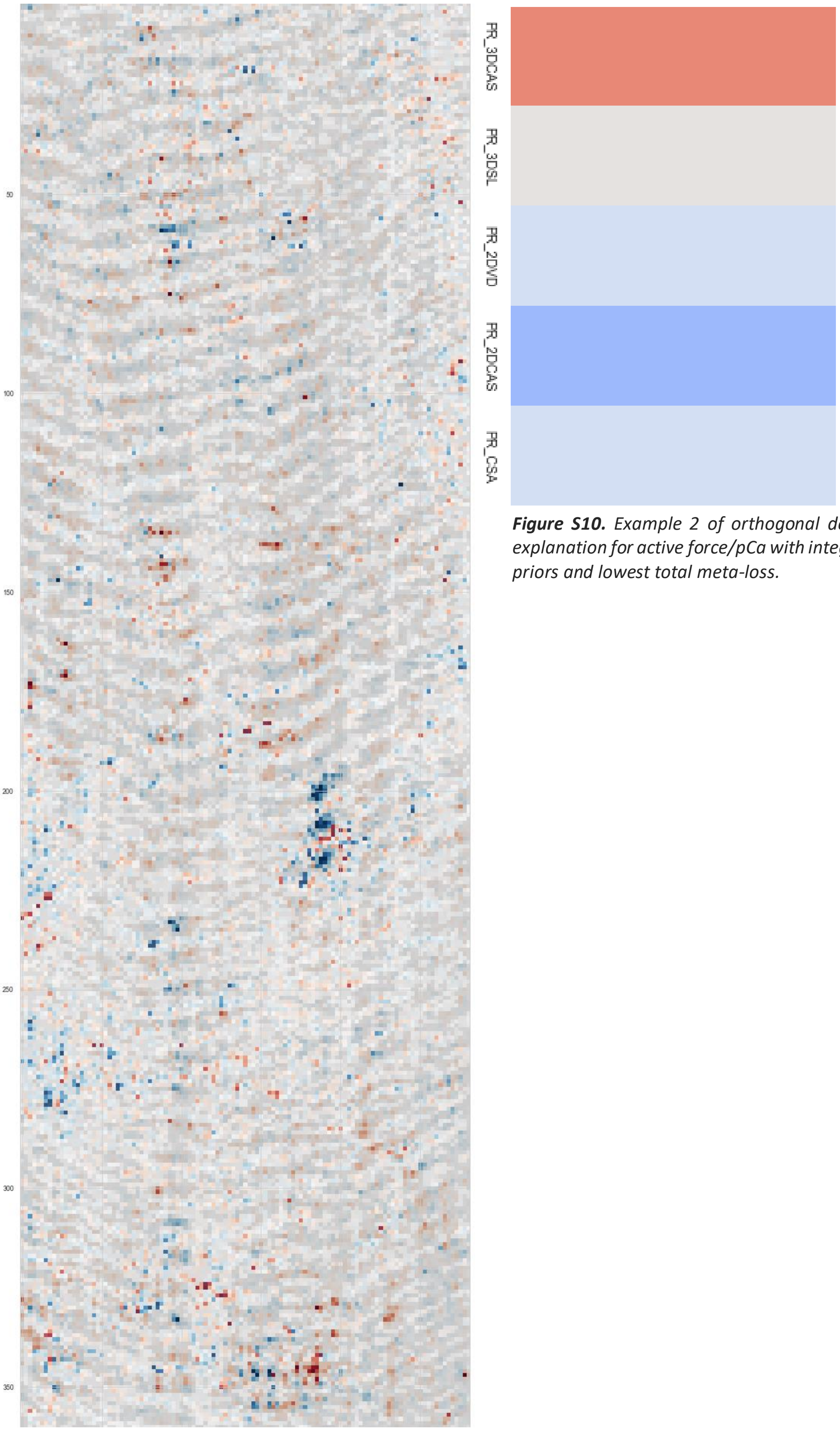

Figure S10. *Example 2 of orthogonal decision explanation for active force/pCa with integrated priors and lowest total meta-loss.*

Task pCa50: Example #1

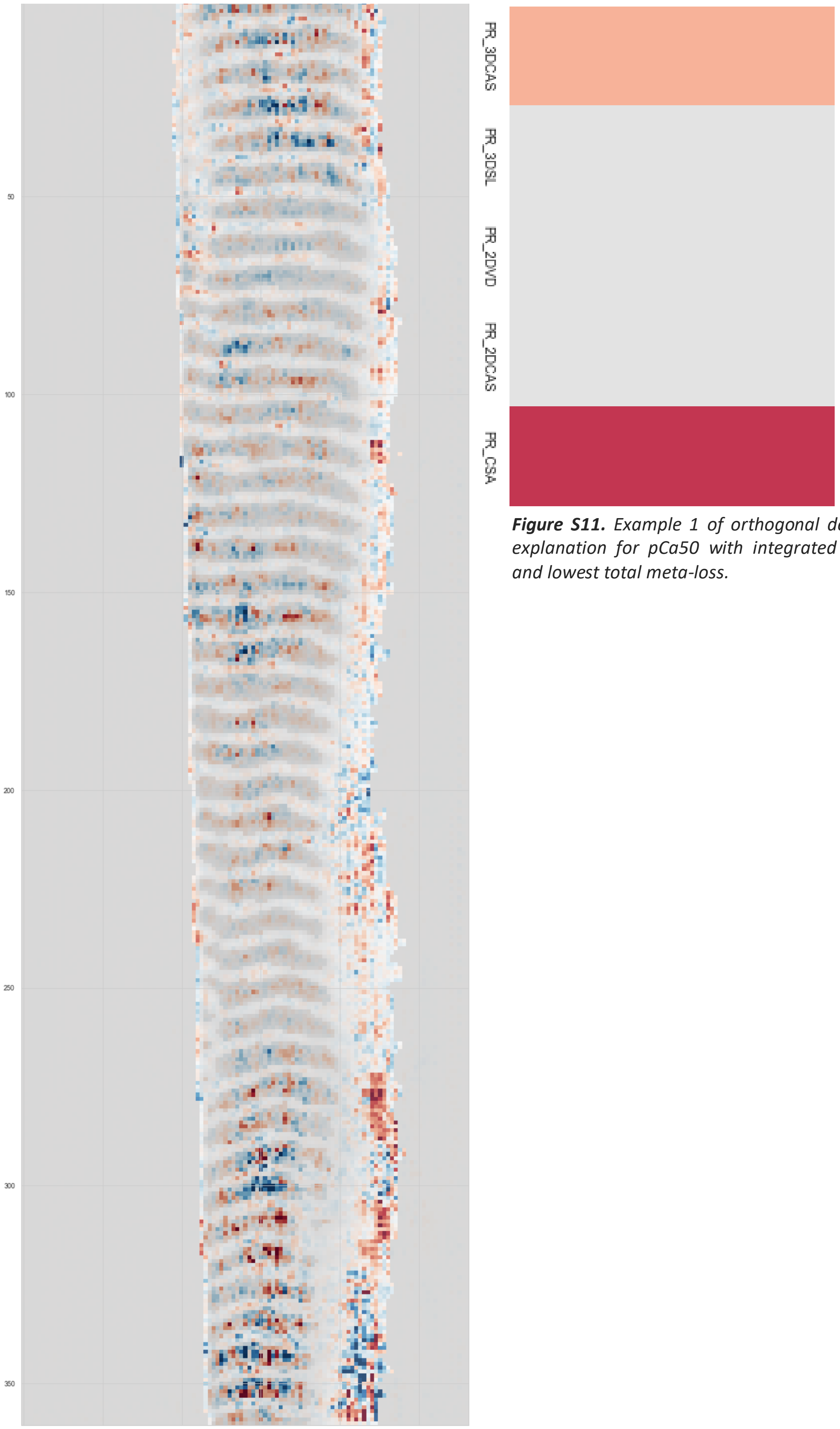

Figure S11. *Example 1 of orthogonal decision explanation for pCa50 with integrated priors and lowest total meta-loss.*

Example #2

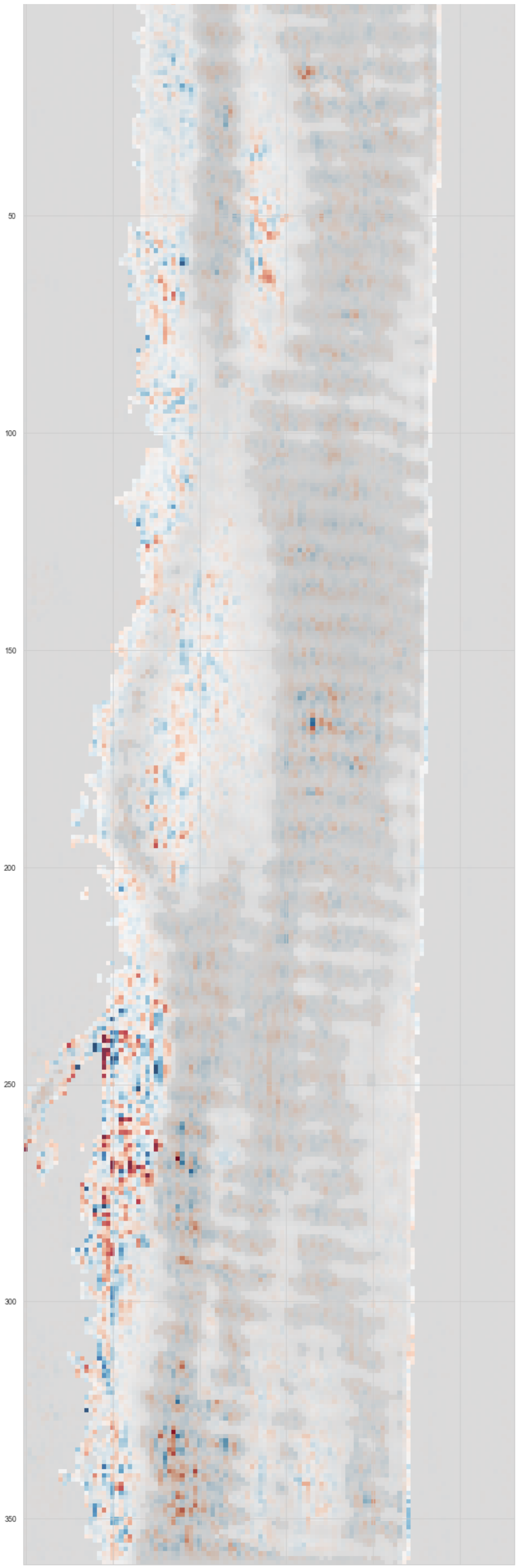

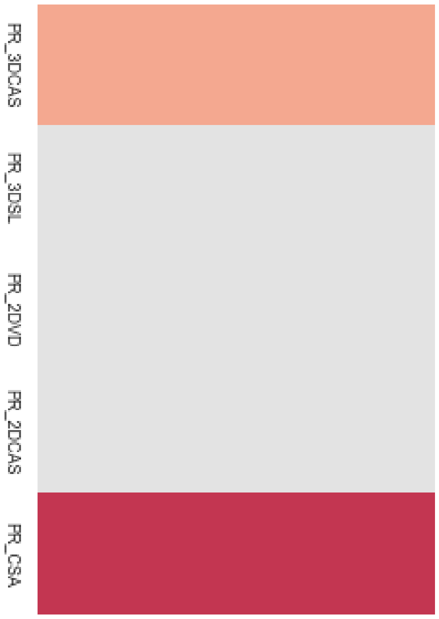

Figure S12. *Example 2 of orthogonal decision explanation for pCa50 with integrated priors and lowest total meta-loss.*
